# Far-field probing of leaky topological states in all-dielectric metasurfaces

**DOI:** 10.1038/s41467-018-03330-9

**Published:** 2018-03-02

**Authors:** Maxim A. Gorlach, Xiang Ni, Daria A. Smirnova, Dmitry Korobkin, Dmitry Zhirihin, Alexey P. Slobozhanyuk, Pavel A. Belov, Andrea Alù, Alexander B. Khanikaev

**Affiliations:** 1 The Department of Electrical Engineering, Grove School of Engineering, City College of the City University of New York, NY, 10031 USA; 2 ITMO University, Saint Petersburg, 197101 Russia; 3 The Graduate Center of the City University of New York, NY, 10016 USA; 4 The Department of Electrical and Computer Engineering, The University of Texas at Austin, Austin, TX 78712 USA; 5 Advanced Science Research Center, City University of New York, New York, NY 10031 USA

## Abstract

Topological phase transitions in condensed matter systems give rise to exotic states of matter such as topological insulators, superconductors, and superfluids. Photonic topological systems open a whole new realm of research and technological opportunities, exhibiting a number of important distinctions from their condensed matter counterparts. Photonic modes can leak into free space, which makes it possible to probe topological photonic phases by spectroscopic means via Fano resonances. Based on this idea, we develop a technique to retrieve the topological properties of all-dielectric metasurfaces from the measured far-field scattering characteristics. Collected angle-resolved spectra provide the momentum-dependent frequencies and lifetimes of the photonic modes that enable the retrieval of the effective Hamiltonian and extraction of the topological invariant. Our results demonstrate how the topological states of open non-Hermitian systems can be explored via far-field measurements, thus paving a way to the design of metasurfaces with unique scattering characteristics controlled via topological effects.

## Abstract (web summary)

Topological modes in photonics systems are not completely confined to the structure but can leak into free space. Here, Gorlach et al. exploit these leaky modes to probe the topological properties of a dielectric metasurface from far-field scattering measurements.

## Introduction

Topological phase transitions in two-dimensional (2D) condensed matter systems have attracted an enormous interest^1–4^ crowned by the Nobel Prize in Physics in 2016. With the advent of photonic topological insulators^5–12^ the research domain expanded to include topological transitions for light^13,14^. However, most of the topological photonic systems considered until today have largely ignored the fact that photonic modes can couple to the continuum of free-space modes, and the exploration of the topological modes has been limited to the near-field properties. Only recently have topological phenomena in photonic systems associated with leaky states attracted attention in the context of non-Hermitian systems^15,16^, including Floquet systems^17,18^ and non-radiative modes in the continuum^19^.

While the leakage of photonic modes to the free-space continuum can be considered as a challenge, as it makes the system non-Hermitian, it may also offer an alternative route to explore the topology of photonic bands with far-field measurements, provided that the radiative decay of the topological modes is controllable and nondestructive. This is especially relevant in the context of metasurfaces—ultrathin photonic structures enabling a variety of novel optical devices, including flat lenses and wave-plates to control polarization and angular momentum of optical beams^20–26^. Endowing metasurfaces with topological properties may open a whole new realm of opportunities in the field of light scattering and wavefront control, as the synthetic gauge field for light have been shown to controllably affect all degrees of freedom associated with electromagnetic radiation^24,27^. Provided the metasurface is engineered to sustain topological photonic modes above the light line, they may couple to the radiative continuum, giving rise to unique scattering features, such as Fano resonances discriminating light by its polarization, angular momentum, or any other synthetic degree of freedom.

In recent years, it has become a traditional approach to look at the topological properties of photonic systems through the prism of the bulk-boundary correspondence principle^28^, focusing on investigation of their edge characteristics, including chiral and helical edge states. Although this strategy to explore topological properties^6,9,29,30^ is quite appealing due to the distinctive features such as backscattering-immune propagation, it can divert us from new and potentially beneficial properties of bulk modes stemming from their topological nature.

To explore these ideas, we design and fabricate a metasurface that supports radiative photonic modes in the near-infrared spectral range and exhibits a topological transition. As the parameters of the metasurface are tuned, the coupling of topological modes to the radiation continuum enables the observation of a topological transition, which is accompanied by the inversion of bright and dark modes, referred to as band crossing. Angle-resolved spectroscopy allows for the direct extraction of spectral positions, intensity, and bandwidth of the peaks corresponding to the excitation of bulk states of the metasurface from the transmission and reflection spectra. As demonstrated below, these parameters provide valuable information about the nature of the eigenmodes supported by the structure, enabling the univocal retrieval of topological invariants from far-field measurements.

## Results

### Topological metasurface

**Fig. 1:**
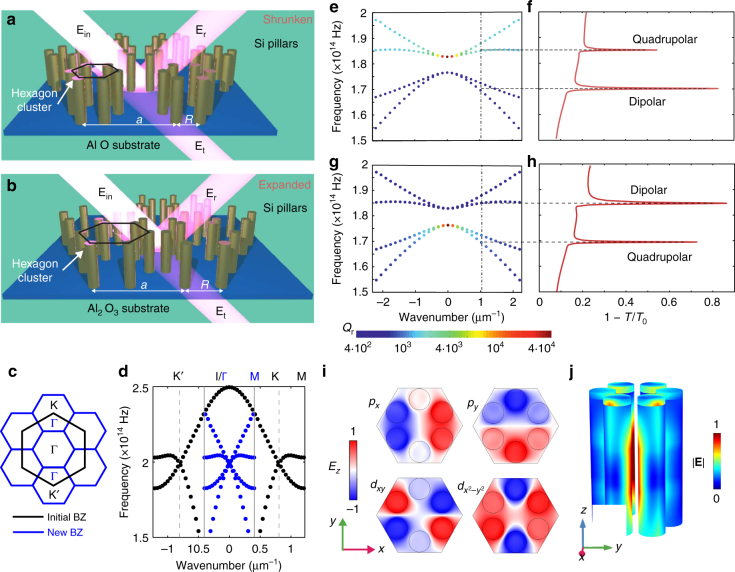
Design of a photonic metasurface exhibiting topological transition and band inversion above the light line. **a**,** b** Geometry of a metasurface based on a triangular lattice of hexamers of silicon pillars on a sapphire (Al_2_O_3_) substrate: **a** shrunken structure with *a*/*R* > 3; **b** expanded structure with *a*/*R* < 3. Here *a* is the lattice constant of distorted lattices. **E**_in_, **E**_r_, and **E**_t_, are incident, reflected and transmitted fields. **c** Brillouin zone of an unperturbed honeycomb lattice *a*/*R* = 3 (black) and the triangular lattice under study *a*/*R* ≠ 3 (blue) obtained by the honeycomb lattice deformation. **d** Band structure of (infinitesimally) shrunk/expanded hexamers exhibits folding (of black dotted bands) near K and K′ points to Γ point in the new Brillouin zone (blue dotted bands). As a result of such symmetry reduction, valleys (pseudo-spins) mix, which leads to a topological transition. **e**–**h** Demonstration of band inversion: Complex photonic band structure for the four doublet bands of **e** shrunken *a*/*R* = 3.15 and **g** expanded *a*/*R* = 2.85, structures. Color encodes the radiative quality factor of the modes. Calculated extinction spectra 1 − *T*/*T*_0_ of the shrunken (**f**) and expanded (**h**) metasurfaces. Spectra are normalized to the transmittance *T*_0_ through Al_2_O_3_ substrate and are computed for fixed tangential wavenumber *k*_||_ = 1.04 × 10^6^ m^−1^. The peaks in far-field spectra correspond to the eigenmode frequencies for the given tangential wavenumber. **i** Simulated field profiles showing the *E*_*z*_ field component at the top surface of the unit cell for dipolar (*p*_*x*_ and *p*_*y*_) and quadrupolar (*d*_*xy*_ and $d_{x^2 - y^2}$) eigenmodes for expanded (topological) structure. **j** Side view of the field distribution in the unit cell under resonant excitation of the dipolar mode for the normal incidence of *x*-polarized light. Electric field magnitude is normalized to the maximum value

The metasurface consists of cylindrical Si pillars arranged into hexagon clusters with edge length *R*, placed at the sites of a triangular lattice with period *a* (Fig. 1a, b). The topological properties of its infinite 2D analog possessing *C*_6v_ symmetry have been the subject of several recent studies^31–37^. For *a*/*R* = 3, this system represents a conventional honeycomb lattice (with the unit cell containing two cylinders), which exhibits Dirac cones centered at K and K′ points of the Brillouin zone. However, if the lattice symmetry is reduced by clustering six neighboring pillars so that $a{\mathrm{/}}R\not = 3$ (distorted lattice), the size of the unit cell increases leading to the reshaping of the first Brillouin zone as shown in Fig. 1c. As a consequence, the Dirac points appear in the vicinity of the Γ point due to the bands folding in the distorted lattice as shown in Fig. 1d. Additionally, the interaction between the valleys of the former honeycomb lattice caused by such symmetry reduction leads to the opening of photonic bandgaps (Fig. 1e, g). This interaction can be viewed as synthetic spin–orbit coupling between two pseudo-spin states corresponding to the former valley degree of freedom of the unperturbed honeycomb lattice. Previous studies suggested that the shrunken structure with *a*/*R* > 3 is topologically trivial, whereas the expanded system with *a*/*R* < 3 is topologically nontrivial^31^ (see also Supplementary Note 1), which, as shown below, remains true for the case of the open system. The important distinction, however, is that in the case of the open metasurface, the folded modes appear within the light cone, and, therefore, are radiatively coupled to the continuum of free space, which enables their far-field characterization^38^ by spectroscopic methods (Fig. 1f, h).

To perform spectroscopy measurements of metasurfaces with two topologically distinct geometries, we fabricated two sets of samples (Fig. 1a, b) with the same lattice constant *a* = 750 nm, radius of the pillars *r* = 75 nm, and height of the pillars *h* = 1000 nm. The sizes of the clusters *R* shown in Fig. 1a, b were chosen to be *a*/*R* = 3.15 and *a*/*R* = 2.85 for shrunken and expanded structures, respectively.

### Effective Hamiltonian

Although the topological properties of the perturbed honeycomb lattice have been previously explored under the tight binding approximation^31^, here we take a different approach, fully considering the electromagnetic nature of the system. Correspondingly, the effective Hamiltonian near the Γ point of the Brillouin zone is obtained directly from Maxwell’s equations by the plane wave expansion method^8,12^ (Supplementary Note 2). To describe the properties of photonic bands of bulk modes in the 2D photonic crystal in the vicinity of the Γ point, we construct an effective 4 × 4 Hamiltonian. Focusing on vertical dipolar modes with *E*_*z*_ component of the electric field directed along the axis of Si rods, we find the results in compliance with the tight binding model (Supplementary Note 1 and refs.^31,32^). The derived effective 4 × 4 Hamiltonian assumes the following form^31^:

1 $$\hat H = \left( {\begin{array}{*{20}{c}} {\hat H_ - } & {\hat K} \\ {\hat K^\dagger } & {\hat H_ + } \end{array}} \right),$$

where the 2 × 2 matrices $\hat H_ \pm$ and $\hat K$ are

2 $$\hat H_ \pm = \left( {\begin{array}{*{20}{c}} {\mu ({\bf{k}})} & {v\left( { \mp k_x - i{\kern 1pt} k_y} \right)} \\ {v{\kern 1pt} \left( { \mp k_x + i{\kern 1pt} k_y} \right)} & { - \mu ({\bf{k}})} \end{array}} \right),$$

3 $$\hat K = \left( {\begin{array}{*{20}{c}} {\alpha {\kern 1pt} \left( {k_x + ik_y} \right)^2} & 0 \\ 0 & { - \alpha {\kern 1pt} \left( {k_x - i{\kern 1pt} k_y} \right)^2} \end{array}} \right),$$

*μ*(**k**) = *μ* + *βk*^2^, *μ* and *β* are the mass term and band parabolicity, respectively. This form of the Hamiltonian corresponds to the basis choice

4 $$\left| \psi \right\rangle = \left( {\left| {p_ - } \right\rangle ,\left| {d_ - } \right\rangle ,\left| {p_ + } \right\rangle ,\left| {d_ + } \right\rangle } \right)^T,$$

where, $\left| {p_ \pm } \right\rangle = p_x \pm i{\kern 1pt} p_y$ are the circularly polarized dipolar modes and $\left| {d_ \pm } \right\rangle = d_{x^2 - y^2} \pm i{\kern 1pt} d_{xy}$ are the circularly polarized quadrupolar modes, which both originate from modes supported by an isolated meta-molecule (the hexamer of 6 dielectric rods).

The four eigenstates of the Hamiltonian Eq. (1) exhibit a pairwise degeneracy at the Γ point *k* = 0 (Supplementary Note 1 and 2), with eigenvalues equal to *μ* (dipolar modes) and −*μ* (quadrupolar modes). Numerically computed field distribution of the modes (shown in Fig. 1i, j) demonstrate in-plane and out-of-plane variation, respectively, and confirm their dipolar and quadrupolar form and strong confinement to the pillars. Transition from shrunken to expanded design is accompanied by the band inversion for dipolar and quadrupolar eigenstates, and by the reversal of the sign of the mass term *μ*. In addition, the degeneracy between left- and right-handed circularly polarized modes is removed for nonzero values of *k* due to the term being proportional to *α*. However, for the topological invariant calculation, this term is inessential and can be dropped^31^. As a result of this approximation, the Hamiltonian splits into two independent 2 × 2 blocks, and the effective pseudo-spin can be introduced. A straightforward calculation^39^ of spin Chern number (Supplementary Note 1) yields

5 $$C = \frac{1}{2}\left[ {{\mathrm{sgn}}{\kern 1pt} {\kern 1pt} \mu - {\mathrm{sgn}}{\kern 1pt} \beta } \right],$$

thus, to evaluate the topological invariant one has to extract the mass term *μ* and the parabolicity of the bands *β* from the experimentally measured spectra.

### Retrieval of topological state via far-field spectroscopy

The modes of interest are located above the light cone, and, for the case of a metasurface of a finite thickness, they couple to the radiation continuum of an open background medium^38^. As a result, the modes acquire a finite lifetime and the corresponding eigenfrequencies become complex-valued. Thus, the silicon pillars constituting the metasurface effectively act as laterally coupled cavities, and the leaky modes of the structure can be excited by the polarization currents induced at the interface of the metasurface by the incident fields. However, taking into consideration the symmetry of the modes, normally incident light can only couple to the dipolar modes, which thus represent super-radiant modes, whereas coupling to the quadrupolar modes is suppressed because of the symmetry mismatch with the incident field, and they represent sub-radiant modes. The quadrupolar modes can still be excited indirectly through coupling to the dipolar modes at oblique incidence, because of the hybridization of dipolar and quadrupolar modes away from the Γ point. This mechanism is referred to as an extrinsic coupling to dark modes in Fano-resonant systems and is caused by the symmetry reduction due to the finite value of the in-plane wave vector components.

Of special interest are the effects of the topological transition on the radiative coupling of the modes to the continuum, which should follow the band inversion described above, giving rise to switching of the bright (dipolar) and dark (quadrupolar) modes, and, thus, enabling control over the far-field scattering properties of the metasurface.

To support our claims, the complex photonic band structures for both the shrunken and expanded metasurfaces were calculated in Comsol Multiphysics with radiative decay fully considered. The results are shown in Fig. 1e, g. Indeed, the dipolar and quadrupolar modes at the Γ point appear to be bright and dark, respectively, as reflected by the radiative quality factors (essentially, the lifetimes of the modes). Even at oblique incidence, when modes are hybridized, the respective bands may have radiative quality factors that differ by orders of magnitude. The numerically calculated extinction spectra at oblique incidence are shown in Fig. 1f, h, and confirm excitation of the two eigenmodes, which manifest as two peaks. The predominantly dipolar and quadrupolar modes can be easily discriminated by their distinct bandwidth and the amplitude of the corresponding peaks. This allows one to establish the symmetry of the mode without the need to look into the near-field pattern and to extract the mass term *μ* needed for evaluation of the topological invariant.

To formalize our description in the context of the topological properties of leaky modes, we develop a unified approach utilizing the temporal coupled mode theory (CMT) along with the electromagnetic effective Hamiltonian description. We write the CMT equations for the amplitudes of the right-handed and left-handed circularly polarized leaky modes coupled to the external source in the following block-diagonal form:

$$- i{\kern 1pt} \varepsilon \left| {\psi _ \pm } \right\rangle = - i{\kern 1pt} \hat H_ \pm \left| {\psi _ \pm } \right\rangle + \chi \left( {\begin{array}{*{20}{c}} {E_{{\mathrm{in}}}} \\ 0 \end{array}} \right) - \left( {\begin{array}{*{20}{c}} {\gamma _0 + \gamma _{\mathrm{r}}} & 0 \\ 0 & {\gamma _0} \end{array}} \right)\left| {\psi _ \pm } \right\rangle , 6$$

where $\left| {\psi _ \pm } \right\rangle = \left( {\left| {p_ \pm } \right\rangle ,\left| {d_ \pm } \right\rangle } \right)^T$ is the mode composed of *p* (dipole) and *d* (quadrupole) eigenmodes of the system. The first term of Eq. (6) describes the evolution of the coupled modes in a closed system, $\chi$ describes the coupling strength of the system to the external field $E_{{\mathrm{in}}} = E_0{\mathrm{/}}\sqrt 2$ (*E*_0_ is the field amplitude), *γ*_0_ represents non-radiative losses in the structure, and $\gamma _{\mathrm{r}} = \chi ^2{\mathrm{/}}2$ describes the radiative losses. The use of this form of dynamic equation can be also justified by applying electromagnetic perturbation theory for an open system as shown in Supplementary Note 4.

Once Eq. (6) is solved, one can obtain expressions for the transmission (and reflection) coefficients:

7 $$t_ \pm = t_0{\kern 1pt} \left[ {1 - \chi \psi _ \pm (p){\mathrm{/}}E_{{\mathrm{in}}}} \right],$$

where *ψ*_±_(*p*) is the first (dipolar) component of the two-component wave function $\left| {\psi _ \pm } \right\rangle$. Since the results are the same for left-handed and right-handed circular polarizations, from now on we omit the ± subscript. Plugging the Hamiltonian into Eq. (7), we derive the expression for the extinction in the form

8 $$\tilde R \equiv 1 - \frac{{\left| t \right|^2}}{{\left| {t_0} \right|^2}} = 2\gamma _0\chi ^2{\textstyle{{[\varepsilon + \mu ({\bf{k}})]^2 + v^2{\kern 1pt} k^2 + \gamma _0^2} \over {\left[ {\mu ({\bf{k}})^2 - \varepsilon ^2 + v^2{\kern 1pt} k^2 + \gamma _0{\kern 1pt} \left( {\gamma _0 + \chi ^2/2} \right)} \right]^2 + \left[ {2\gamma _0{\kern 1pt} \varepsilon + \chi ^2/2{\kern 1pt} (\mu ({\bf{k}}) + \varepsilon )} \right]^2}}}.$$

Equation (8) suggests in particular that two peaks in the $\tilde R$ spectrum can be observed due to excitation of dipolar and quadrupolar eigenmodes. Thus, the frequency of the most pronounced peak at incidence close to normal is around *f*_0_ + *μ*, whereas the frequency of the less intense peak is found around *f*_0_ − *μ*, where *f*_0_ is the mid-gap frequency.

Overall, there are six parameters in the effective model that control the entire dispersion and topological properties of the far-field response: *μ*, $\gamma _{\mathrm{r}} = \chi ^2{\mathrm{/}}2$, *γ*_0_, *v*, and *β* (see Eqs. (2) and (6)). The numerical values of parameters comprising the effective Hamiltonian can be obtained by fitting the experimental data. Next, by using the developed technique, we analyze the experimental data for both shrunken (*a*/*R* = 3.15) and expanded (*a*/*R* = 2.85) structures and extract their topological characteristics.

**Fig. 2:**
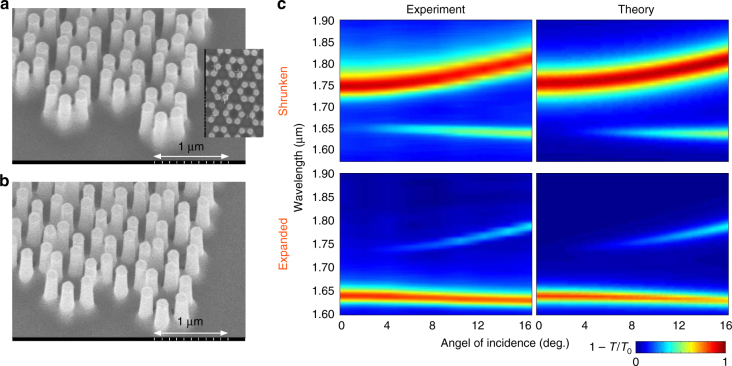
Measured extinction spectra for the fabricated dielectric metasurface. **a** Scanning electron microscopy image of the fabricated shrunken and expanded structures with *a*/*R* = 3.15 (**a**) and *a*/*R* = 2.85 (**b**), respectively, lattice period *a* = 750 nm, pillar height *h* = 1000 nm, and pillar radius *r* = 75 nm. **c** Experimental vs numerical spectra for the fabricated shrunken and expanded samples with *a*/*R* = 3.15 and *a*/*R* = 2.85, respectively. Color encodes the magnitude of $\tilde R = 1 - T{\mathrm{/}}T_0$ for *p*-polarized incident light

To confirm our theoretical predictions, a set of shrunk and expanded silicon samples on top of sapphire (Al_2_O_3_) substrates was fabricated and tested experimentally. The structures were illuminated using the halogen source Ocean Optics HL-2000-LL. The transmission was measured for both structures by the spectrometer Ocean Optics NIR Quest NQ 512–2.2 in the range of the wavelengths from 896 to 2142 nm corresponding to the frequency range (1.40 ÷ 3.35) × 10^14^ Hz. The measured transmittance was normalized to the one of a sapphire substrate. Figure 2 shows the color map of the quantity $\tilde R = 1 - T{\mathrm{/}}T_0$ as a function of the wavelength and the incidence angle for both the shrunken and expanded structures.

In the wavelength range from 1600 to 1900 nm, two peaks are clearly observed. The detailed spectroscopy results for the whole studied spectral range are provided in Supplementary Note 3. In agreement with our numerical calculations, the experimental data clearly show that the most intense and broad peak for the shrunken structure is the low-frequency one, whereas for the expanded structure the high-frequency peak is more pronounced. Thus, the shrunken structure is characterized by a negative effective mass *μ*, while the expanded structure is described by a positive effective mass *μ*.

**Fig. 3:**
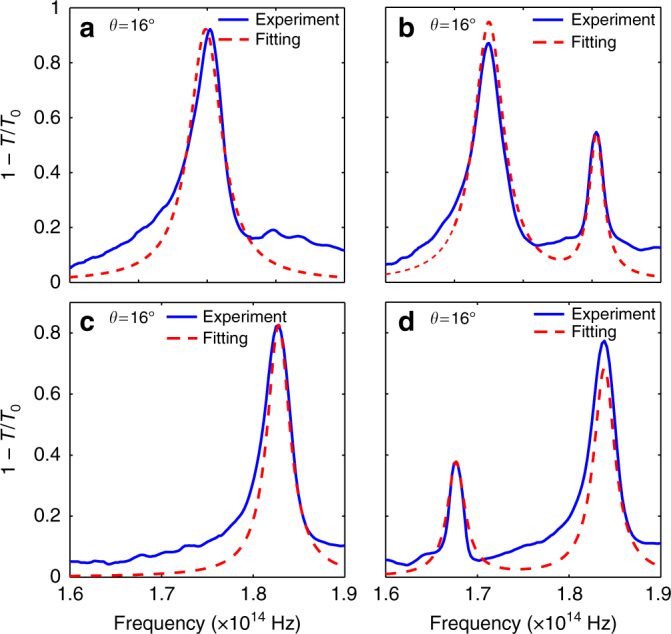
Extraction of parameters of the effective Hamiltonian by fitting. The experimental extinction spectra (1 − *T*/*T*_0_, where *T*_0_ is the substrate transmittance) are fitted by the analytical model Eq. (8) for **a**, **b** shrunken and **c**, **d** expanded structures with *a*/*R* = 3.15 and *a*/*R* = 2.85, respectively. Fitting for two different angles of incidence **a**, **c** *θ* = 0 and **b**, **d** *θ* = 16° is shown

Next, we applied the above analytical model to fit the measured data with Eqs. (6) and (8), as detailed in the Supplementary Note 3. The results of the simplest fitting algorithm for the two particular angles of incidence, 0° and 16°, are illustrated in Fig. 3 for the cases of both shrunken and expanded structures. The extracted parameters of the effective Hamiltonian are listed in Table 1. Importantly, these numbers appear to be nearly independent of the fitting algorithm (see details in Supplementary Note 3). From the measured transmittance spectra, we recover again that the shrunken structure is topologically trivial, since the effective mass *μ* and parabolicity parameter *β* have the same sign, yielding zero spin Chern number. On the contrary, the expanded structure appears to be topologically nontrivial, due to the opposite signs of *μ* and *β*, yielding a spin Chern number of 1. It is important to mention that for non-Hermitian systems (including the one considered here) the definition of topological invariant has been a subject of active discussion^15–18,39^. In Supplementary Note 7, following ref. ^39^, we show that the topological invariant remains unaffected by the presence of loss for the case of the metasurface studied here.

**Table 1:** Parameters of the effective Hamiltonian extracted by fitting the experimental data

Structure	*μ* (THz)	*β* (m^2^ s^−1^)	*v* (10^6^ ms^−1^)	*γ*_r_ (THz)	*γ*_0_ (THz)	*C* (Spin Chern number)
Shrunken, *a*/*R* = 3.15	−5.22	−1.00	4.76	1.83	1.03	0
Expanded, *a*/*R* = 2.85	5.22	−1.96	7.31	0.437	1.06	1

Interestingly, the topological properties have been directly extracted previously from the bulk spectrum for 1D systems only^40^. A different approach based on the edge properties was taken by Mittal et al.^41^, who introduced a unit quantum flux at the edge and observed a shift in the position of the edge spectrum. Our results prove for the first time that it is possible to retrieve the topological properties of an open 2D system via far-field measurements in a robust and direct way.

### Far-field and near-field observation of edge states

**Fig. 4:**
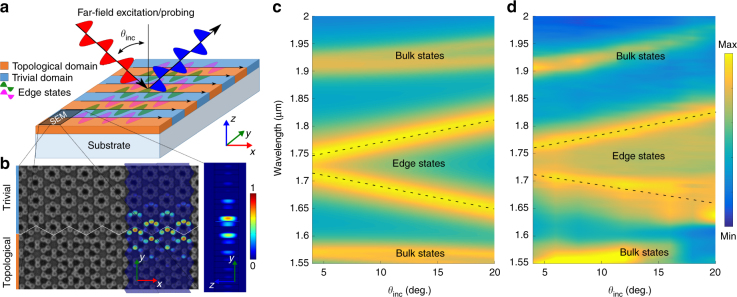
Far-field spectroscopy of topological edge states. **a** Schematic optical setup for far-field mapping of photonic bands corresponding to edge states in a topological meta-grating. Meta-grating is formed by stitching 12 unit cells of alternating topological/expanded (orange) and trivial/shrunken (light blue) domains of the metasurface with 30 domain walls in total. **b** SEM image of the proximity to one of the domain walls overlaid with the computed field profile (electromagnetic energy density) of the edge state. **c** The results of CMT calculations of the scattering from topological meta-grating. Periodic conditions are imposed on the outer interfaces. **d** Measured far-field reflectance spectra revealing edge states within the bulk bandgap. The parameters used in CMT model are: on-site energy *ω*_0_ = 173.61 THz, intra-cell tunneling amplitude *K* = 33.00 THz, inter-cell tunneling amplitude *J* = 23.00 THz, radiative loss *γ*_r_ = 0.25 THz, and non-radiative loss *γ*_0_ = 0.30 THz

In order to confirm that the structure under study indeed supports topological edge modes, we have performed additional experimental studies of the domain wall formed between the two topologically distinct structures (shrunken and expanded) and further visualized edge modes with the far-field and near-field experimental techniques. The far-field experimental studies have been performed on a sample consisting of an array of domain walls formed by repetitive stitching of 12 unit cells of shrunken and expanded domains along a zig-zag cut of the lattice. The schematic of the experimental setup is shown in Fig. 4a with an SEM image of the sample near the domain wall in Fig. 4b. The results of numerical calculations performed within the use of the CMT are presented in Fig. 4c and reveal the presence of the edge states within the bandgap of bulk modes. The examination of numerically computed field profiles of the edge modes overlaid with the SEM image confirms their localization at the domain wall as shown in false-color plot in Fig. 4b. The experimental far-field measurements of sample’s reflectance are presented in Fig. 4b and confirm the presence of edge states within the topological bandgap. Interestingly, despite the presence of the additional scattering channels due to diffraction, the modes have relatively narrow bandwidth and they can be easily distinguished from the bulk modes by their distinctive linear (1D Dirac-like) dispersion at small tangential wavenumbers. Thus, the leakage of the edge states appears to be strong enough so that they can be excited and measured in far-field region, yet, the non-Hermiticity it introduces is not strong enough to destroy the topological state of the system. Thus, the presence of the edge states in our system serves as another piece of evidence of the topological properties of the structure despite its open nature.

Additional proof-of-concept experimental near-field studies of the edge modes were conducted in the microwave spectral range. Note that, due to the dielectric nature of the structure and scalability of Maxwell’s equations, the obtained results can be directly transferred to the structures operating in the optical domain.

We considered a metasurface consisting of dielectric rods arranged into the lattice with the permittivity of the rods *ε* = 10, lattice period *a* = 37.5 mm, radius and height of dielectric rods are *r* = 1.5 mm and *h* = 10 mm, respectively. The cluster size *R* (Fig. 1a, b) was chosen such that *a*/*R* = 3.33 and *a*/*R* = 2.73 for shrunken and expanded structures, respectively. For these parameters, the bandgap is characterized by the mid-gap frequency of 6.79 GHz with a total bandwidth of 310 MHz.

**Fig. 5:**
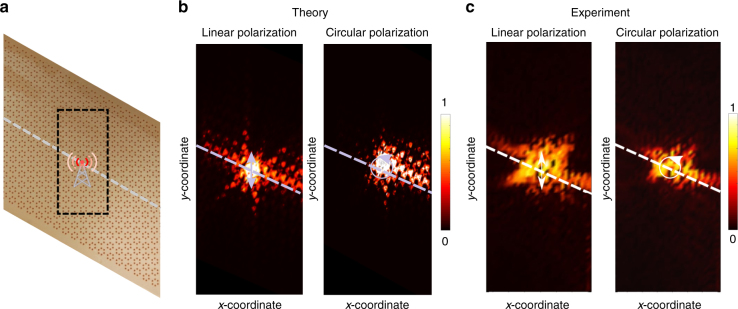
Proof-of-concept experiment observation of pseudo-spin polarized edge states in microwaves. **a** Photograph of fabricated metasurface. The gray dashed line indicates the position of the domain wall and the black dashed rectangle shows the spatial *xy* range of near-field scanning shown in **b**, **c**. The antenna indicates the position of the dipole source. **b** Numerically calculated near-field of the edge states excited by linearly and circularly polarized dipoles. **c** Color map of the measured near-field of the edge states at the frequency *f* = 6.84 GHz for the linearly and circularly polarized excitations. The unidirectional excitation by the circularly polarized field confirms pseudo-spin locking of the edge states

The experimental sample was constructed by embedding commercially available dielectric powder (Eccostock HiK with the permittivity *ε* = 10 and loss tangent tan *δ* = 0.0007) (Ceramic powder datasheet https://www.eccosorb.com/products-eccostock-hik-powder.htm) into the machine-processed substrate made from Styrofoam material (permittivity of 1.15 and negligible losses) in which the holes were drilled (Fig. 5a). All the measurements were performed in an anechoic chamber using an automatic mechanical near-field scanning setup and an electric field probe connected to the receiving port of the vector network analyzer (Agilent E8362C). The probe was oriented normally with respect to the interface of the structure. The near-field was scanned at 2 mm distance from the back interface of the metasurface to avoid a direct contact between the probe and the sample. To excite the edge state, the subwavelength dipole source whose polarization could be adjusted from circular to linear was placed next to the domain wall in the center of the expanded unit cell. Figure 5b shows the results of full wave simulations of the field distribution of the edge state. The corresponding experimental map presented in Fig. 5c clearly shows that the mode excited inside the bandgap at frequency *f* = 6.84 GHz is indeed localized to the domain wall and represents the edge state. To confirm the topological properties of the edge states, we performed near-field excitation both by linearly and circularly polarized dipole fields. As can be seen from Fig. 5b, c, the linearly polarized dipole leads to bi-directional excitation of the edge state. In contrast, the circularly polarization excitation leads to one-way propagation of the edge state evidencing the locking of the polarization and the propagation direction, thus, confirming the topological nature of the corresponding electromagnetic mode^42,43^.

These results prove independently that the metasurface considered here exhibits a topological transition when its design is changed from shrunken to expanded structure, thus validating the introduced far-field measurement technique for the detection of a topological transition in open photonic systems such as metasurfaces.

## Discussion

In this paper, we have introduced the concept of topological metasurfaces and demonstrated that their scattering characteristics, radiative quality factors of the modes in particular, can be controlled by synthetic gauge fields. The developed formalism allows us to relate topological properties and the far-field scattering, thus enabling the extraction of the effective Hamiltonian and the topological invariant (pseudo-spin Chern number) of the metasurface from the measured transmission/reflection spectra. Coupling of photonic modes of the structure to the free-space modes allows for the direct probing and visualization of the topological phase transitions in the open photonic system. We believe that our results open a new avenue for designing metasurfaces with desirable scattering properties controllable via their topological characteristics. Provided the properties of metasurfaces are designed to stem from edge states, their response may exhibit topological robustness to continuous modifications and disorder, which can be of immense value for practical applications.

## Methods

### Modeling and calculations

To derive the effective Hamiltonian and topological properties of the photonic crystal, we use the tight binding approach (Supplementary Note 1), as well as the plane wave expansion method (Supplementary Note 2). To calculate the far-field scattering properties of the structure, we employ the CMT (Supplementary Note 3). Perturbative analysis of the radiative losses for the metasurface is performed by the guided mode expansion method (Supplementary Note 4). The calculation of scattering from the topological meta-grating based on the CMT is detailed in Supplementary Note 6. The role of non-Hermiticity and topological invariants in the presence of radiative losses are discussed in Supplementary Note 7.

### Sample fabrication

A scheme of the fabrication procedure is outlined in Supplementary Note 5 and Supplementary Fig. 6.

### Data availability

The data that support the findings of this study are available from the corresponding author upon request.

## Electronic supplementary material

Supplementary Information
