# Supplementary material for: Far-field probing of leaky topological states in all-dielectric metasurfaces
Source: Nat Commun. 2018 Mar 2;9:909. doi: 10.1038/s41467-018-03330-9 (PMC5834506; doi:10.1038/s41467-018-03330-9)
Supplement: Supplementary file 1 — Supplementary Information [file 41467_2018_3330_MOESM1_ESM.pdf]

Supplementary Information: Far-field probing of leaky topological states in all-dielectric metasurfaces

Maxim A. Gorlach[1,2,*], Xiang Ni[1,3,*], Daria A. Smirnova[1], Dmitry Korobkin[1], Dmitry Zhirihin[2], Alexey P. Slobozhanyuk[2], Pavel A. Belov[2], Andrea Alù[4] & Alexander B. Khanikaev[1,2,3]

[1]*The Department of Electrical Engineering, Grove School of Engineering, City College of the City University of New York, New York 10031, USA*

[2]*ITMO University, Saint Petersburg 197101, Russia*

[3]*The Graduate Center of the City University of New York, New York, 10016, USA*

[4]*The Department of Electrical and Computer Engineering, The University of Texas at Austin, Austin, Texas 78712, USA*

Supplementary Note 1. Effective Hamiltonian from the tight-binding model

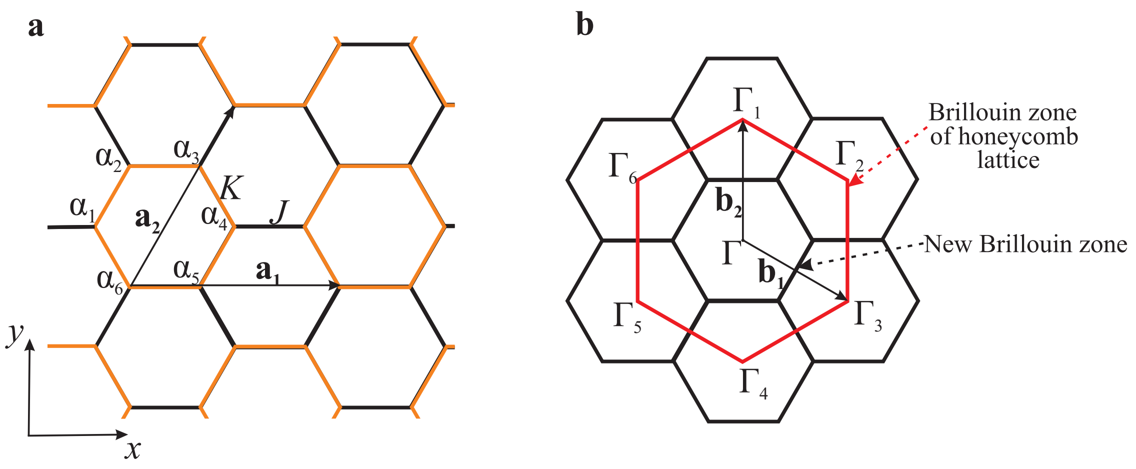

Supplementary Figure 1. Top view of the structure under study and its Brillouin zones. (a) A schematic representation of triangular lattice formed by hexamer clusters with inter- and intracell tunneling amplitudes equal to J (black lines) and K (orange lines), respectively. α_i, $i = 1..6$ represent Wannier functions. (b) First Brillouin zone of the lattice.

In this section, we develop a tight binding description of the system under study (Sup. Fig. 1) assuming coupling only between the neighboring resonators. We deduce an effective 4×4 Hamiltonian and evaluate the topological invariant for the system partially reproducing the results of Refs. [1,2].

In the chosen coordinate frame, the translation vectors of the lattice are equal to $\mathbf{a}_1 = a\ (1, 0)$ and $\mathbf{a}_2 = a\ \left(1/2, \sqrt{3}/2\right)$ [Sup. Fig. 1(a)], where a is the lattice period. Reciprocal vectors are $\mathbf{b}_1 = G\ \left(\sqrt{3}/2, -1/2\right)$, $\mathbf{b}_2 = G\ (0, 1)$, where $G = 4\,\pi/(\sqrt{3}\,a)$. The first Brillouin zone of the lattice is a hexagon with the area three times larger than that of a simple honeycomb lattice, Sup.

Fig. 1(b). Under tight binding approximation the equations describing the system are as follows:

$$\hat{H}\ |\psi\rangle = \varepsilon\ |\psi\rangle\ , \tag{1}$$

where the six-component wave function is written in Wannier basis and reads

$$|\psi\rangle = (\alpha_1, \alpha_2, \alpha_3, \alpha_4, \alpha_5, \alpha_6)^T\ , \tag{2}$$

and the Hamiltonian is equal to

$$\hat{H} = \begin{pmatrix} 0 & -K & 0 & -J\,e^{-i\,\mathbf{k}\mathbf{a}_1} & 0 & -K \\ -K & 0 & -K & 0 & -J\,e^{i\mathbf{k}(\mathbf{a}_2-\mathbf{a}_1)} & 0 \\ 0 & -K & 0 & -K & 0 & -J\,e^{i\mathbf{k}\mathbf{a}_2} \\ -J\,e^{i\mathbf{k}\mathbf{a}_1} & 0 & -K & 0 & -K & 0 \\ 0 & -J\,e^{i\mathbf{k}(\mathbf{a}_1-\mathbf{a}_2)} & 0 & -K & 0 & -K \\ -K & 0 & -J\,e^{-i\mathbf{k}\mathbf{a}_2} & 0 & -K & 0 \end{pmatrix} \tag{3}$$

Eigenvalue equation Sup. Eq. (1) defines the dispersion law for the excitations propagating in the lattice. The dispersion of the structure six eigenmodes for different ratios of the tunneling amplitudes J/K and $k_x = 0$ is illustrated in Sup. Fig. 2. It is seen that for $J = K$ there is a fourfold degeneracy point in the spectrum (Dirac point). The difference between J and K partially lifts the degeneracy and leads to the opening of bandgap. In the general case, the energy spectrum contains two singlet states and four doublet states. Doublet states have pairwise degeneracy at Γ point. The degeneracy is lifted for nonzero values of k.

Our goal is to deduce the effective Hamiltonian describing the doublet bands of the system in the vicinity of Γ point. Singlet states should be excluded from the consideration while their interaction with the doublet states should be properly incorporated into the effective Hamiltonian.

Quite naturally, the form of the effective Hamiltonian depends on the choice of the basis in Hilbert space. We choose the basis formed by the eigenmodes of the isolated unit cell composed of

six identical resonators. These eigenmodes can be found as eigenvectors of the Hamiltonian from Sup. Eq. (3) with $J = 0$.

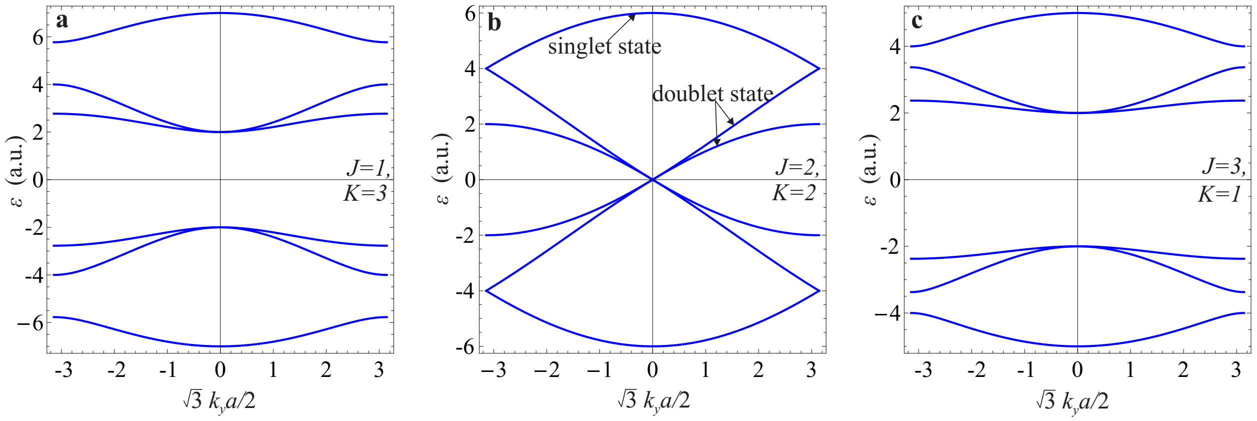

Supplementary Figure 2. **Dispersion of excitations in the system under study for $k_x = 0$.** (a) $J = 1$, $K = 3$; (b) $J = 2$, $K = 2$; (c) $J = 3$, $K = 1$: a bandgap closing and reopening in the vicinity of Γ point is observed.

$$|u_1\rangle = 1/\sqrt{6}\ (-1, 1, -1, 1, -1, 1)^T\ , \tag{4}$$

$$|u_2\rangle = 1/\sqrt{6}\ (1, 1, 1, 1, 1, 1)^T\ , \tag{5}$$

$$|u_3\rangle = 1/\sqrt{6}\ \left(1, e^{i\pi/3}, e^{2i\pi/3}, -1, e^{-2i\pi/3}, e^{-i\pi/3}\right)^T\ , \tag{6}$$

$$|u_4\rangle = 1/\sqrt{6}\ \left(1, e^{2i\pi/3}, e^{-2i\pi/3}, 1, e^{2i\pi/3}, e^{-2i\pi/3}\right)^T\ , \tag{7}$$

$$|u_5\rangle = 1/\sqrt{6}\ \left(1, e^{-i\pi/3}, e^{-2i\pi/3}, -1, e^{2i\pi/3}, e^{i\pi/3}\right)^T\ , \tag{8}$$

$$|u_6\rangle = 1/\sqrt{6}\ \left(1, e^{-2i\pi/3}, e^{2i\pi/3}, 1, e^{-2i\pi/3}, e^{2i\pi/3}\right)^T\ . \tag{9}$$

The obtained eigenmodes can be classified by their behavior with respect to the symmetry transformation of the cluster, i.e. $\pi/3$ rotation of the hexamer around z axis perpendicular to its plane. Un-

der such symmetry transformation vectors $u_{1,2}$ multiply by ∓ 1, vectors $|u_{3,5}\rangle$ transfrom as $x \mp iy$, $|u_{4,6}\rangle$ transform as $(x \mp iy)^2$. Thus, vectors $|u_{1,2}\rangle$ describe singlet states, $|u_{3,5}\rangle$ describe dipole $|p_{\mp}\rangle$ modes, and $|u_{4,6}\rangle$ correspond to quadrupole $|d_{\mp}\rangle$ modes.

If one simply excludes the first two columns and two rows of the obtained 6×6 Hamiltonian matrix corresponding to singlet states, an effective Hamiltonian for p and d modes will only be correct up to the terms linear in k. However, as it will be demonstrated below, terms proportional to k^2 are important for the correct evaluation of the topological invariant. Therefore, we take into account the interaction of singlet states with p and d modes using degenerate perturbation theory and calculate the effective 4×4 Hamiltonian as follows [3]:

$$H^{(\mathrm{eff})}_{mm'} = H_{mm'} - \frac{1}{2} \sum_s \left[\frac{1}{E^0_s - E^0_m} + \frac{1}{E^0_s - E^0_{m'}} \right] H'_{ms}\, H'_{sm'}\ , \tag{10}$$

where the initial system Hamiltonian $\hat{H}$ is represented as a sum of diagonal part $\hat{H}_0$ [in our case $\hat{H}(k = 0)$] and perturbation $\hat{H}'$ [in our case $\hat{H}'$ contains terms proportional to k]. H'_{ms} thus represents the matrix elements between doublet states of interest labelled by indices m and singlet states excluded from the consideration and labelled by the indices s. We apply a unitary transformation

$$W = i \begin{pmatrix} \sigma_z & 0 \\ 0 & \sigma_z \end{pmatrix}\ , \tag{11}$$

to the obtained 4×4 matrix and for notation simplicity redefine k_x as k_y and k_y as k_x. The effective Hamiltonian incorporating terms proportional to k and k^2 reads:

$$\hat{H}_{\mathrm{eff}} = \begin{pmatrix} \mu(\mathbf{k}) & v\,(k_x - i\,k_y) & \alpha\,(k_x + i\,k_y)^2 & 0 \\ v\,(k_x + i\,k_y) & -\mu(\mathbf{k}) & 0 & -\alpha\,(k_x - i\,k_y)^2 \\ \alpha\,(k_x - i\,k_y)^2 & 0 & \mu(\mathbf{k}) & v\,(-k_x - i\,k_y) \\ 0 & -\alpha\,(k_x + i\,k_y)^2 & v\,(-k_x + i\,k_y) & -\mu(\mathbf{k}) \end{pmatrix}\ , \tag{12}$$

where $\mu(\mathbf{k}) = \mu + \beta\, k^2$,

$$v = \sqrt{3}\, J/2\,, \tag{13}$$

$$\mu = J - K\,, \tag{14}$$

$$\beta = -\frac{3J\,(J+K)}{4\,(2J+K)}\,, \tag{15}$$

$$\alpha = \frac{3\,J\,K}{8\,(2J+K)}\,. \tag{16}$$

Effective Hamiltonian Sup. Eq. (12) provides rather accurate description of the eigenmode dispersion in the vicinity of Γ point. Note that the term $\propto \alpha$ associated with the coupling of left- and right circularly polarized eigenmodes captures the effect of degeneracy lifting for doublet bands for nonzero values of k. The comparison of the results obtained from the approximate Hamiltonian Sup. Eq. (12) with those derived from the full lattice model Sup. Eq. (3) is provided in Sup. Fig. 3.

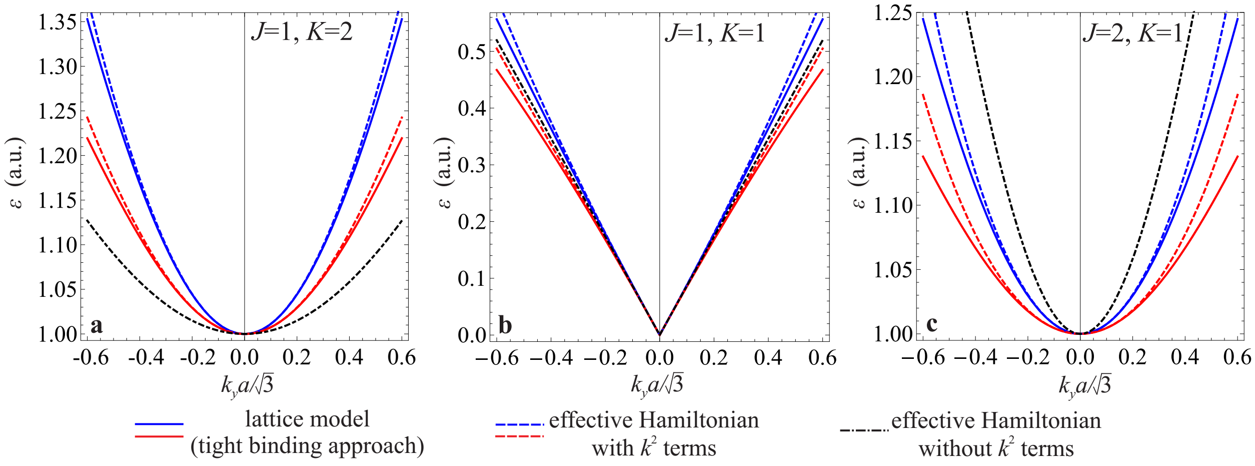

Supplementary Figure 3. Validation of the approximate formulas for the effective Hamiltonian for $k_x = 0$. Solid lines show the results of the full lattice model, dashed lines (dot-dashed line) are plotted by diagonalizing the effective Hamiltonian with (without) k^2 terms. k^2 terms are needed in order to explain lifting of the degeneracy of doublet bands for nonzero k. (a) $J = 1$, $K = 2$; (b)

$J = K = 1$; (c) $J = 2$, $K = 1$.

As a next step, we evaluate the topological invariant for the Hamiltonian Sup. Eq. (12). In such calculation, the term $\propto \alpha$ can be neglected [1] and as a result the Hamiltonian splits into two decoupled blocks

$$\hat{H} = \begin{pmatrix} \hat{H}_- & 0 \\ 0 & \hat{H}_+ \end{pmatrix} , \tag{17}$$

where 2×2 blocks $\hat{H}_\pm$ are given by the formula

$$\hat{H}_\pm = \begin{pmatrix} \mu + \beta\, k^2 & v\,(\mp k_x - i\, k_y) \\ v\,(\mp k_x + i\, k_y) & -\mu - \beta\, k^2 \end{pmatrix} , \tag{18}$$

and correspond to the two circular polarizations of eigenmodes. Two possible polarizations can be associated with the pseudospin degree of freedom and thus spin Chern number $C = (C_- - C_+)/2$ can be introduced, where $C_\pm$ is Chern number for the individual block. Each of the blocks Sup. Eq. (18) has the structure of the Dirac Hamiltonian and the calculation of Chern number is straightforward [4]. Finally we obtain

$$C = \frac{1}{2}\left[\text{sgn}\,\mu - \text{sgn}\,\beta\right] = \frac{1}{2}\left[\text{sgn}\,(J-K) + 1\right] . \tag{19}$$

Thus, the tight binding approach predicts that shrunken structure with $a/R > 3$ and $J < K$ is trivial ($C = 0$), whereas expanded structure with $a/R < 3$ and $J > K$ is topological ($C = 1$) in accordance with Ref. [1]. Supplementary Equation (19) suggests also that if $\beta\, k^2$ correction to the effective mass is neglected, one will not be able to distinguish topological and trivial regimes.

Supplementary Note 2. Effective Hamiltonian from the plane wave expansion method

The results of Supplementary Note 1 were obtained in the limiting case when only nearest neighbor coupling is essential and the field is predominantly localized inside the individual resonators.

In order to demonstrate generality of our results and to prove the applicability of the effective Hamiltonian Sup. Eq. (12) to the experimental structure, we analyze an opposite limiting case when there is no pronounced field localization in the individual resonator and the eigenmodes of the system can be approximated by the plane waves. The structure consists of dielectric cylinders with the permittivity that differs only slightly from the permittivity of the background medium. Under such assumptions it is also possible to obtain analytical expressions for the effective Hamiltonian using plane wave expansion method [5,6]. We consider a TM polarization of the wave with the wave vector perpendicular to the axis of cylinder, Oz. Permittivity of all cylinders along their axes is $\varepsilon_{zz} \equiv \varepsilon_r$. The equation for E_z component of electric field has a form

$$\left[q^2\,\varepsilon(x,y) + \partial_x^2 + \partial_y^2\right]\, E_z(x,y) = 0\,, \tag{20}$$

where $q = \omega/c$, $\varepsilon(x,y) = \varepsilon_r$ inside cylinder and $\varepsilon(x,y) = 1$ outside of the cylinder. Further we expand the field $E_z(x,y)$ and the permittivity $\varepsilon(x,y)$ in Fourier series as follows:

$$E_z(\mathbf{r}) = \sum_G E_{\mathbf{G}}\, e^{i(\mathbf{G}+\mathbf{k})\cdot\mathbf{r}}\,, \tag{21}$$

$$\varepsilon(\mathbf{r}) = \sum_G \varepsilon_{\mathbf{G}}\, e^{i\mathbf{G}\cdot\mathbf{r}}\,, \tag{22}$$

where $\mathbf{G}$ and $\mathbf{G}'$ denote reciprocal lattice vectors. Combining Sup. Eqs. (21), (22) with Sup. Eq. (20), we obtain:

$$q^2 \sum_{G'} \varepsilon_{\mathbf{G}-\mathbf{G}'}\, E_{\mathbf{G}'} - \left[(G_x + k_x)^2 + (G_y + k_y)^2\right]\, E_{\mathbf{G}} = 0\,. \tag{23}$$

Now we consider the system of equations in the vicinity of Γ point of the crystal first Brillouin zone [see Sup. Fig. 1(b)]. We truncate the system leaving only the contributions from Γ_0 and six neighboring Γ points denoted by the indices 1-6 in Sup. Fig. 1(b). The length of all reciprocal lattice vectors $\mathbf{G_i}$ is equal to $G = 4\pi/(\sqrt{3}\,a)$, where $a = 2\,R + R'$ is the lattice period, R is the edge length for a hexagonal cluster, and R' is the distance between the centers of the nearest rods from the different clusters. The radius of the rod is denoted by r.

First we calculate the Fourier coefficients $\varepsilon_{\mathbf{G}-\mathbf{G}'}$ comprising the system Sup. Eqs. (23). By definition,

$$\varepsilon_{\mathbf{G}} = \frac{1}{S_0} \int\limits_{S_0} \varepsilon(x,y)\, e^{-i\mathbf{G}\cdot\mathbf{r}}\, d^2\vec{r}\,, \tag{24}$$

where $S_0 = a^2\,\sqrt{3}/2$ is the area of the structure unit cell. For instance, $\varepsilon_{\mathbf{G_0}} \equiv \varepsilon_0$ is defined by

$$\varepsilon_0 = 1 + \frac{6\pi\,(\varepsilon_r - 1)\,r^2}{S_0}\,. \tag{25}$$

Other coefficients comprising the truncated system Sup. Eqs. (23) are as follows: $\varepsilon_1 = \varepsilon_{\mathbf{G_1}} = \varepsilon_{\mathbf{G_2}} = \cdots = \varepsilon_{\mathbf{G_6}}$. The expression for ε_1 reads:

$$\varepsilon_1 = -\frac{4\pi^2 r^2\,(\varepsilon_r - 1)}{\sqrt{3}\,S_0}\,\frac{2\,J_1(Gr)}{Gr}\,\frac{R - R'}{a} \approx -\frac{4\pi^2 r^2\,(\varepsilon_r - 1)}{\sqrt{3}\,S_0}\,\frac{R - R'}{a} \equiv -u\,, \tag{26}$$

where J_1 is the Bessel function of the first kind and an assumption $Gr \ll 1$ is used. If ε_r is real, the quantity ε_1 is also purely real. $\varepsilon_3 = \varepsilon_{\mathbf{G_3}-\mathbf{G_6}} = \varepsilon_{\mathbf{G_2}-\mathbf{G_5}} = \varepsilon_{\mathbf{G_1}-\mathbf{G_4}}$. Under the assumption $G\,r \ll 1$ ε_3 is given by the formula

$$\varepsilon_3 = 2u\,. \tag{27}$$

Quite importantly, the coefficient u vanishes in the case of a simple honeycomb lattice thus being analogous to the parameter $J - K$ in tight binding model. $\varepsilon_2 = \varepsilon_{\mathbf{G_3}-\mathbf{G_1}} = \varepsilon_{\mathbf{G_5}-\mathbf{G_3}} = \varepsilon_{\mathbf{G_1}-\mathbf{G_5}} =$

$\varepsilon_{\mathbf{G_4}-\mathbf{G_2}} = \varepsilon_{\mathbf{G_6}-\mathbf{G_4}} = \varepsilon_{\mathbf{G_2}-\mathbf{G_6}}$. This coefficient is also purely real:

$$\varepsilon_2 = -\frac{3\pi r^2\,(\varepsilon_r - 1)}{S_0}\,\frac{2\,J_1(Gr\sqrt{3})}{Gr\sqrt{3}} \approx -\frac{3\pi r^2\,(\varepsilon_r - 1)}{S_0}\,, \tag{28}$$

The truncated system (23) yields the set of the self-consistent equations with respect to the amplitudes $E_i \equiv E_{\mathbf{G_i}}$:

$$\left[\varepsilon_0 - q^{-2}\,k^2\right]\,E_0 + \varepsilon_1\,E_1 + \varepsilon_1\,E_2 + \varepsilon_1\,E_3 + \varepsilon_1\,E_4 + \varepsilon_1\,E_5 + \varepsilon_1\,E_6 = 0\,, \tag{29}$$

$$\varepsilon_1\,E_0 + \left[\varepsilon_0 - q^{-2}\,\left(G^2 + 2Gk_y + k^2\right)\right]\,E_1 + \varepsilon_1\,E_2 + \varepsilon_2\,E_3 + \varepsilon_3\,E_4 + \varepsilon_2\,E_5 + \varepsilon_1\,E_6 = 0\,, \tag{30}$$

$$\varepsilon_1\,E_0 + \varepsilon_1\,E_1 + \left[\varepsilon_0 - q^{-2}\,\left(G^2 + G\sqrt{3}k_x + Gk_y + k^2\right)\right]\,E_2 + \varepsilon_1\,E_3 + \varepsilon_2\,E_4 + \varepsilon_3\,E_5 + \varepsilon_2\,E_6 = 0\,, \tag{31}$$

$$\varepsilon_1\,E_0 + \varepsilon_2\,E_1 + \varepsilon_1\,E_2 + \left[\varepsilon_0 - q^{-2}\,\left(G^2 + G\sqrt{3}k_x - G\,k_y + k^2\right)\right]\,E_3 + \varepsilon_1\,E_4 + \varepsilon_2\,E_5 + \varepsilon_3\,E_6 = 0\,, \tag{32}$$

$$\varepsilon_1\,E_0 + \varepsilon_3\,E_1 + \varepsilon_2\,E_2 + \varepsilon_1\,E_3 + \left[\varepsilon_0 - q^{-2}\,\left(G^2 - 2G\,k_y + k^2\right)\right]\,E_4 + \varepsilon_1\,E_5 + \varepsilon_2\,E_6 = 0\,, \tag{33}$$

$$\varepsilon_1\,E_0 + \varepsilon_2\,E_1 + \varepsilon_3\,E_2 + \varepsilon_2\,E_3 + \varepsilon_1\,E_4 + \left[\varepsilon_0 - q^{-2}\,\left(G^2 - G\sqrt{3}\,k_x - G\,k_y + k^2\right)\right]\,E_5 + \varepsilon_1\,E_6 = 0 \tag{34}$$

$$\varepsilon_1\,E_0 + \varepsilon_1\,E_1 + \varepsilon_2\,E_2 + \varepsilon_3\,E_3 + \varepsilon_2\,E_4 + \varepsilon_1\,E_5 + \left[\varepsilon_0 - q^{-2}\,\left(G^2 - G\sqrt{3}\,k_x + G\,k_y + k^2\right)\right]\,E_6 = 0\,, \tag{35}$$

where $k^2 = k_x^2 + k_y^2$. The matrix of this system is Hermitian. To simplify the analysis, we perform a unitary transformation of the system matrix as follows:

$$U = \frac{1}{\sqrt{3}}\begin{pmatrix} \sqrt{3} & 0 & 0 & 0 & 0 & 0 & 0 \\ 0 & 0 & 1 & 0 & 1 & 0 & 1 \\ 0 & 1 & 0 & 1 & 0 & 1 & 0 \\ 0 & 0 & 1 & 0 & \eta^2 & 0 & \eta \\ 0 & 0 & 1 & 0 & \eta & 0 & \eta^2 \\ 0 & 1 & 0 & \eta^2 & 0 & \eta & 0 \\ 0 & 1 & 0 & \eta & 0 & \eta^2 & 0 \end{pmatrix}\,, \tag{36}$$

where $\eta = e^{2\pi\,i/3}$. After the transformation Sup. Eq. (36) the matrix of the system consists of 3×3 and 4×4 blocks, the coupling between these blocks is proportional to k. However, the blocks are not diagonal even for $k = 0$.

As a first step, we diagonalize the matrix of the system for $k = 0$ and small u (from now

on terms proportional to u^2, u^3, etc. are neglected). The necessary transformation is given by the matrix $W = W_1 \oplus W_2$, where matrix W_1

$$W_1 = \begin{pmatrix} 1 & -\sqrt{3}\,u/(q^{-2}\,G^2 - 2\varepsilon_2) & -\sqrt{3}\,u/(q^{-2}\,G^2 - 2\varepsilon_2) \\ 0 & -1/\sqrt{2} & 1/\sqrt{2} \\ \sqrt{6}\,u/(q^{-2}G^2 - 2\varepsilon_2) & 1/\sqrt{2} & 1/\sqrt{2} \end{pmatrix} \tag{37}$$

diagonalizes the 3×3 block of the Hamiltonian for $k = 0$ with the precision up to the terms linear in u, and the matrix W_2

$$W_2 = \frac{1}{\sqrt{2}} \begin{pmatrix} 0 & i & 0 & e^{i\pi/6} \\ e^{5i\pi/6} & 0 & e^{i\pi/6} & 0 \\ e^{5i\pi/6} & 0 & e^{-5i\pi/6} & 0 \\ 0 & -i & 0 & e^{i\pi/6} \end{pmatrix} . \tag{38}$$

diagonalizes the 4×4 block of the Hamiltonian for $k = 0$.

Next we would like to exclude the 3×3 block of singlet states. To this end we treat coupling between the two blocks of the matrix using degenerate perturbation theory in the special form [3]. Similarly to the tight binding treatment we also make a redefinition $k_x \to k_y$ and $k_y \to k_x$. As a result, we obtain the following eigenvalue problem:

$$\hat{H}_{\text{eff}}\ |\psi\rangle = \lambda\ |\psi\rangle\ . \tag{39}$$

Here, the effective 4×4 Hamiltonian is given by the expression

$$\hat{H}_{\text{eff}} = \begin{pmatrix} \mu + \beta\,k^2 & k_x - i\,k_y & \alpha\,(k_x + i\,k_y)^2 & 0 \\ k_x + i\,k_y & -\mu - \beta\,k^2 & 0 & -\alpha\,(k_x - i\,k_y)^2 \\ \alpha\,(k_x - i\,k_y)^2 & 0 & \mu + \beta\,k^2 & -k_x - i\,k_y \\ 0 & -\alpha\,(k_x + i\,k_y)^2 & -k_x + i\,k_y & -\mu - \beta\,k^2 \end{pmatrix} \tag{40}$$

with $\mu = 3uq^2/G$, $\beta = -Gu/(3\,\varepsilon_2^2\,q^2)$, $\alpha = -G/(3\varepsilon_2\,q^2)$, and $\lambda = -G + q^2\,(\varepsilon_0 - \varepsilon_2)/G - k^2/G - Gk^2/(3\,q^2\,\varepsilon_2)$. The components of bispinor $|\psi\rangle$ comprising Sup. Eq. (39) are defined in

terms of the field Fourier components:

$$\psi_1 = \frac{1}{\sqrt{6}} \left[e^{i\pi/6} E_{\mathbf{G1}} + e^{i\pi/2} E_{\mathbf{G2}} + e^{5i\pi/6} E_{\mathbf{G3}} + e^{-5i\pi/6} E_{\mathbf{G4}} + e^{-i\pi/2} E_{\mathbf{G5}} + e^{-i\pi/6} E_{\mathbf{G6}} \right] , \quad (41)$$

$$\psi_2 = \frac{1}{\sqrt{6}} \left[e^{i\pi/6} E_{\mathbf{G1}} + e^{5i\pi/6} E_{\mathbf{G2}} + e^{-i\pi/2} E_{\mathbf{G3}} + e^{i\pi/6} E_{\mathbf{G4}} + e^{5i\pi/6} E_{\mathbf{G5}} + e^{-i\pi/2} E_{\mathbf{G6}} \right] , \quad (42)$$

$$\psi_3 = \frac{1}{\sqrt{6}} \left[e^{-5i\pi/6} E_{\mathbf{G1}} + e^{5i\pi/6} E_{\mathbf{G2}} + e^{i\pi/2} E_{\mathbf{G3}} + e^{i\pi/6} E_{\mathbf{G4}} + e^{-i\pi/6} E_{\mathbf{G5}} + e^{-i\pi/2} E_{\mathbf{G6}} \right] , \quad (43)$$

$$\psi_4 = \frac{1}{\sqrt{6}} \left[e^{i\pi/6} E_{\mathbf{G1}} + e^{-i\pi/2} E_{\mathbf{G2}} + e^{5i\pi/6} E_{\mathbf{G3}} + e^{i\pi/6} E_{\mathbf{G4}} + e^{-i\pi/2} E_{\mathbf{G5}} + e^{i\pi/6} E_{\mathbf{G6}} \right] . \quad (44)$$

Note that the derived form of the effective Hamiltonian Sup. Eq. (40) is consistent with Sup. Eq. (12) obtained within tight binding approach. The difference between the two methods is only in the identification of parameters μ, β and α. Similarly to the tight binding model $\mu < 0$ corresponds to the shrunken structure ($R < R'$) and $\mu > 0$ describes expanded structure ($R > R'$). However, in contrast to the tight binding description which assumes parameter β negative, this approach predicts that β switches its sign together with μ during the topological transition.

Overall, we recover the same expression for the effective Hamiltonian [Sup. Eqs. (12) and (40)] in the two extreme scenarios. On that basis we use this form of the effective Hamiltonian while analyzing the experimental spectra.

Supplementary Note 3. Coupled mode theory and experimental data fitting

In this section, we discuss a procedure of the topological invariant extraction from the measured extinction spectra. To this end we develop an analytical model for the metasurface extinction based on coupled mode theory [7,8]. We use the effective Hamiltonian in the block-diagonal form Sup. Eq. (17), (18) and employ the following coupled mode equation:

$$- i\,\varepsilon\,|\psi_\pm\rangle = -i\,\hat{H}_\pm\,|\psi_\pm\rangle + \varkappa\begin{pmatrix} E_{\text{in}} \\ 0 \end{pmatrix} - \begin{pmatrix} \gamma_0 + \varkappa^2/2 & 0 \\ 0 & \gamma_0 \end{pmatrix}|\psi_\pm\rangle \tag{45}$$

Here, $|\psi_\pm\rangle$ is the "wavefunction" composed of p (dipole) and d (quadrupole) modes of the system: $|\psi_\pm\rangle = (|p_\pm\rangle\,, |d_\pm\rangle)^T$. $\pm$ sign refers to the left- or right-hand circular polarizations of the mode. The first term of Sup. Eq. (45) describes the evolution of the coupled modes in a closed system. The second term is associated with the external driving field. The third term captures the effect of losses that lead to the diminishing of the mode amplitude including both radiative $\gamma_{\text{r}} = \varkappa^2/2$ and non-radiative γ_0 losses. An explicit expression for $\hat{H}_\pm$ is given by Sup. Eq. (18).

Once Sup. Eq. (45) is solved with respect to the unknown $|\psi_\pm\rangle$, the transmitted wave can be calculated as follows:

$$E_{\text{t}}^{(\pm)} = t_0\,E_{\text{in}}^{(\pm)} + c\,\psi_\pm(p)\,, \tag{46}$$

where t_0 is the transmission coefficient of the sapphire substrate on which the structure is fabricated and $\psi_\pm(p)$ is the first (dipole) component of the two-component wave function $|\psi_\pm\rangle$. The magnitude of the c coefficient is determined by the requirement of energy conservation: the change of the mode energy measured by $\langle\psi_\pm|\,\psi_\pm\rangle$ should be equal to the intensity of the incoming wave minus the intensity of the transmitted and reflected waves and minus the non-radiative dissipation rate. Since the results turn out to be the same for left- and right circular polarizations, we omit the $\pm$ subscript thus obtaining the result:

$$t = t_0\,\left[1 - \varkappa\,\psi(p)/E_{\text{in}}\right]\,. \tag{47}$$

Plugging Sup. Eq. (18) into the Sup. Eq. (47), we derive

$$\tilde{R} \equiv 1 - \frac{|t|^2}{|t_0|^2} = 2\,\gamma_0\,\varkappa^2\,\frac{[\varepsilon + \mu(\mathbf{k})]^2 + v^2\,k^2 + \gamma_0^2}{\left[\mu^2(\mathbf{k}) - \varepsilon^2 + v^2\,k^2 + \gamma_0\,\left(\gamma_0 + \varkappa^2/2\right)\right]^2 + \left[2\gamma_0\,\varepsilon + \varkappa^2/2\,(\mu(\mathbf{k}) + \varepsilon)\right]^2}\,. \tag{48}$$

Supplementary Equation (48) suggests in particular that the peaks in the $1 - T/T_0$ spectrum are impossible without both radiative and non-radiative losses. Overall, there are six parameters in the effective Hamiltonian: μ, $\gamma_{\rm r} = \varkappa^2/2$, γ_0, v and β. To determine these parameters we used the extinction spectra measured in the wavelength range from 896 to 2142 nm for the set of the incidence angles from 0° to 16° with the step equal to 2°. Parameters μ, $\gamma_{\rm r}$ and γ_0 were determined from the spectra for normal incidence ($k = 0$) since in such scenario these three parameters and the center-of-bandgap frequency f_0 are the only essential parameters determining the extinction spectrum [see Sup. Eq. (48)]. The remaining two parameters, v and β, were determined from the extinction spectra for oblique incidence. While analyzing the experimental data on extinction, we aimed to achieve the best possible fit of the spectral positions, width and height of the characteristic peaks in $1-T/T_0$ spectrum (see Sup. Fig. 4). The results of fitting are presented both for shrunken and expanded structures in Sup. Fig. 4.

The deviations of experimental spectra from the analytical formulas occur due to the structure imperfections (defects, non-cylindrical form of pillars, etc.) as well as due to the approximate nature of the used theoretical model. Specifically, the developed analytical approach does not describe the asymmetry of the transmittance peaks observed in experiment. This asymmetry can be explained by the phase difference between the light reflected from sapphire substrate and from Si pillars which gives rise to Fano-type interference of the two reflected waves. The discussed phase difference can be incorporated into our analytical formula by means of the auxiliary parameter φ:

$$t = t_0 \left[e^{i\varphi} - \varkappa\, \psi(p)/E_{\rm in} \right] \ . \qquad (49)$$

Supplementary Figure 4(c,f) shows the influence of the φ parameter on the asymmetry of transmittance peaks. Since incorporation of the additional φ parameter does not change significantly the numerical results for the remaining parameters but makes the numerical procedure of the data

fitting less robust, we prefer to use the simplified formulas Sup. Eqs. (47)-(48).

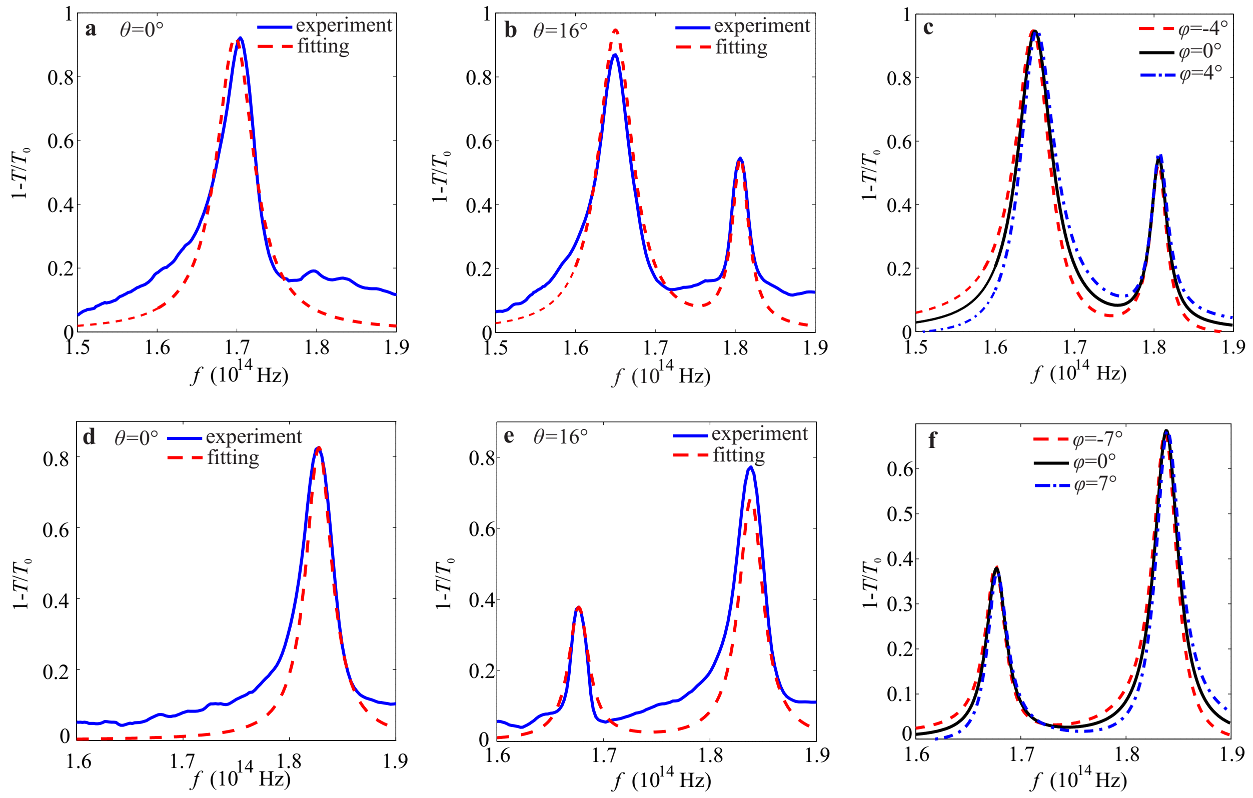

Supplementary Figure 4. Fitting of the experimental extinction spectra $1 - T/T_0$ (T_0 is the substrate transmittance) by the analytical model. Results for shrunken (a-c) and expanded (d-f) structures with $a/R = 3.15$ and $a/R = 2.85$, respectively. (a,b) Normal incidence. (c,d) Incidence angle $\theta = 16°$. (e,f) The influence of the phase shift φ on the asymmetry of the transmittance peaks in shrunken and expanded structures, respectively.

With the developed technique, we analyzed the experimental data for both shrunken ($a/R = 3.15$) and expanded ($a/R = 2.85$) structures for the range of incidence angles from 0 to 16 deg (Sup. Fig. 5). The obtained values of the effective Hamiltonian parameters are provided in the article main text. Using Eq. (5), we calculated spin Chern number for both of the structures confirming

the theoretical result that the shrunken structure is topologically trivial, whereas the expanded one is topological.

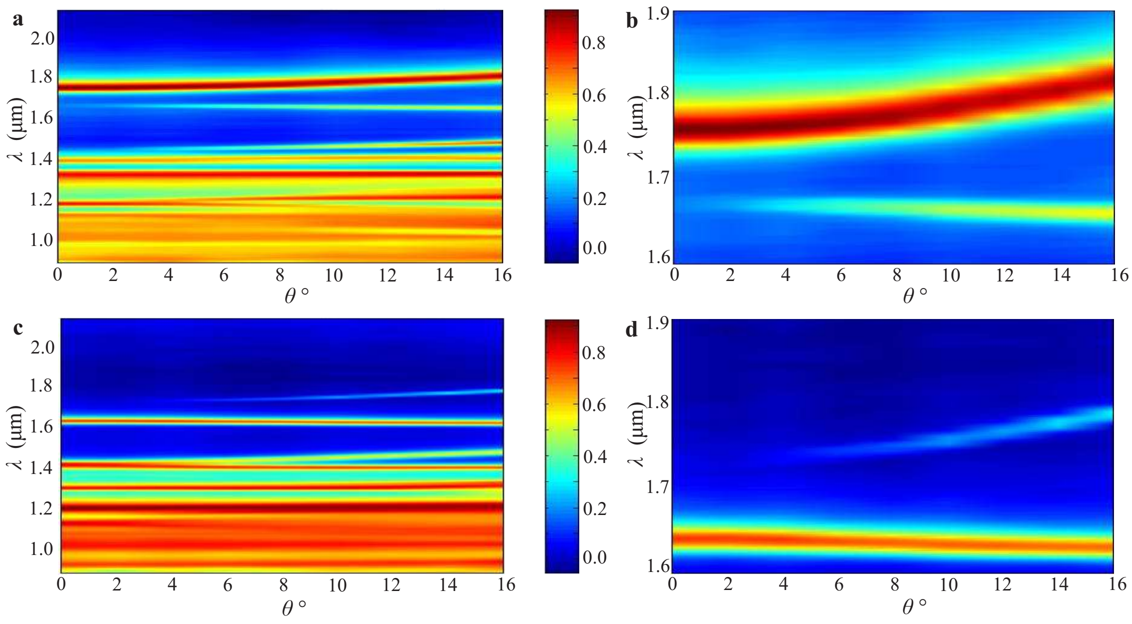

Supplementary Figure 5. Measured extinction spectra for the fabricated dielectric metasurface. Color encodes the magnitude of extinction $1 - T/T_0$ (T and T_0 are metasurface and substrate transmittances, respectively) for p-polarized incident light. (a,b) Results for the shrunken structure with $a/R = 3.15$: (a) whole studied spectral range; (b) range $1600 < \lambda < 1900$ nm. (c,d) Results for the expanded structure with $a/R = 2.85$: (c) whole studied spectral range; (d) range $1600 < \lambda < 1900$ nm.

Supplementary Note 4. Perturbative electromagnetic theory of radiative losses

To support our phenomenological coupled mode approach and elaborate more on the underlying physics, we additionally apply the method of guided mode expansion to describe the photonic bands of the non-Hermitian photonic crystal slab. In particular, this treatment allows us to get an

approximate analytical expression for the radiative coupling. After the folding due to the permittivity modulation, the localized guided modes of the unperturbed dielectic waveguide fall above the cladding light line, leading to a radiative decay and complex eigenfrequencies. To tackle this leaky behavior, we utilize the approach recently developed in Refs. [9–11].

We write the equation for the magnetic field $\mathbf{H}$:

$$\nabla \times \left[\frac{1}{\varepsilon(\mathbf{r})} \nabla \times \mathbf{H}(\mathbf{r}) \right] = q^2 \mathbf{H}(\boldsymbol{r}), \tag{50}$$

where $q = \omega/c$, and focus on the TM-like polarization characterized by the components (H_x, H_y, E_z). To develop a perturbation theory, we adopt the expansion in the basis of Bloch waves. The high-order waves and radiative waves are assumed to be excited by the basic waves. As follows from Maxwell's equations, in our geometry coupling of the waves is governed by the two physical mechanisms: in-plane permittivity modulation and the surface coupling stemming from the permittivity discontinuity at the slab interfaces. The latter effect can be incorporated in equations as boundary conditions using the formalism of Dirac δ-functions.

We employ the basis of Bloch waves $\mathbf{V}$ associated with the reciprocal vectors $\mathbf{G}_j$, $j = 1 \div 6$. In this way, at the Γ point, the components $H_{x,y}$ can be expanded as

$$H_x = \sum_{j=1}^{6} \frac{G_{jy}}{G} V_j \Theta_0(z)\, e^{-i\mathbf{G}_j \cdot \mathbf{r}_\perp} \tag{51}$$

$$H_y = \sum_{j=1}^{6} -\frac{G_{jx}}{G}\, V_j \Theta_0(z)\, e^{-i\mathbf{G}_j \cdot \mathbf{r}_\perp}\ , \tag{52}$$

where $\mathbf{r}_\perp = (x, y)$, $\Theta_0(z)$ is the unperturbed transverse profile of the mode supported by the effective dielectric waveguide, $\int \Theta_0^*(z)\Theta_0(z)dz = 1$. At the Γ point, the basic waves have the same transverse profile $\Theta_0(z)$ and wavenumber β_0. The dispersion relation for TM guided modes

is given in the following implicit form

$$\tan\left(\sqrt{\varepsilon_{\text{avg}}q^2-\beta^2}h\right)=\frac{2\varepsilon_{\text{avg}}\varepsilon_{\text{cl}}\sqrt{(\varepsilon_{\text{avg}}q^2-\beta^2)(\varepsilon_{\text{cl}}q^2-\beta^2)}}{\varepsilon_{\text{cl}}^2(\varepsilon_{\text{avg}}q^2-\beta^2)-\varepsilon_{\text{avg}}^2(\varepsilon_{\text{cl}}q^2-\beta^2)}, \tag{53}$$

where ε_{avg} is the average dielectric permittivity of the slab, and ε_{cl} are the permittivities of the claddings. We assume the permittivities of the upper and lower cladding equal. Here, for simplicity, we neglect TE-TM coupling and coupling to higher-order modes. Thereby, we disregard the H_z component (responsible for coupling to TE modes) that means the trivial transversality condition of the form $G_{jx}H_{jx}+G_{jy}H_{jy}=0$. We also expand the permittivity in Fourier series $1/\varepsilon(\mathbf{r})=\kappa_0(z)+\sum\limits_{\mathbf{G}_j}\kappa_{\mathbf{G}_j}e^{-i\mathbf{G}_j\cdot\mathbf{r}_\perp}$.

Using these expansions, equations for the field components can be recast to the eigenvalue problem for the modes amplitudes

$$(q^2-\beta_0^2)\mathbf{V}=\hat{C}\mathbf{V} \tag{54}$$

where $\mathbf{V}=[V_1,V_2,V_3,V_4,V_5,V_6]^T$. The coupling matrix $\hat{C}$ consists of two parts

$$\hat{C}=\hat{C}_1+\hat{C}_{\text{rad}}\,, \tag{55}$$

where $\hat{C}_1$ and $\hat{C}_{\text{rad}}$ correspond to the direct couplings between basic modes and the coupling with the radiative mode (H_{0x},H_{0y}), respectively. The matrix elements depend on the parameters of the structure.

Due to the $C_{6\mathrm{v}}$ symmetry of the PC slab, the matrix $\hat{C}$ exhibits the following form

$$\hat{C}_1 = \begin{pmatrix} 0 & \kappa_1(G^2+I/2) & \kappa_2(G^2-I/2) & \kappa_3(G^2-I) & \kappa_2(G^2-I/2) & \kappa_1(G^2+I/2) \\ \kappa_1(G^2+I/2) & 0 & \kappa_1(G^2+I/2) & \kappa_2(G^2-I/2) & \kappa_3(G^2-I) & \kappa_2(G^2-I/2) \\ \kappa_2(G^2-I/2) & \kappa_1(G^2+I/2) & 0 & \kappa_1(G^2+I/2) & \kappa_2(G^2-I/2) & \kappa_3(G^2-I) \\ \kappa_3(G^2-I) & \kappa_2(G^2-I/2) & \kappa_1(G^2+I/2) & 0 & \kappa_1(G^2+I/2) & \kappa_2(G^2-I/2) \\ \kappa_2(G^2-I/2) & \kappa_3(G^2-I) & \kappa_2(G^2-I/2) & \kappa_1(G^2+I/2) & 0 & \kappa_1(G^2+I/2) \\ \kappa_1(G^2+I/2) & \kappa_2(G^2-I/2) & \kappa_3(G^2-I) & \kappa_2(G^2-I/2) & \kappa_1(G^2+I/2) & 0 \end{pmatrix} \quad (56)$$

where $\kappa_{1,2,3} = \kappa_{\mathbf{G_1},\mathbf{G_1}-\mathbf{G_3},\mathbf{G_1}-\mathbf{G_4}}$, $I = \int_{PC}\left(-\frac{\partial^2}{\partial z^2} + [\delta(z-h/2)-\delta(z+h/2)]\frac{\partial}{\partial z}\right)\Theta_0(z)\Theta_0^*(z)dz$.

Coupling to free-space modes causes the out-of-plane diffraction losses. The profile of the radiative mode (H_{0x}, H_{0y}) generated by basic waves can be found using the Green's function method [10,11]:

$$H_{0x} = \kappa_1\left(V_1 + \frac{1}{2}V_2 - \frac{1}{2}V_3 - V_4 - \frac{1}{2}V_5 + \frac{1}{2}V_6\right)\tilde{I}_0\,,$$
$$H_{0y} = \kappa_1\left(-\frac{\sqrt{3}}{2}V_2 - \frac{\sqrt{3}}{2}V_3 + \frac{\sqrt{3}}{2}V_5 + \frac{\sqrt{3}}{2}V_6\right)\tilde{I}_0\,,$$

where $\tilde{I}_0$ stands for the integral

$$\tilde{I}_0 = \int_{PC}\left(\frac{\partial^2}{\partial z'^2} - [\delta(z'-h/2)-\delta(z'+h/2)]\frac{\partial}{\partial z'}\right)G_0(z,z')\Theta_0(z')dz'\,,$$

and Green's function is the solution of equation

$$\left(q^2 + \kappa_0\partial^2/\partial z^2 + [(\varepsilon_{\mathrm{cl}}^{-1} - \kappa_0)\{\delta(z-h/2)-\delta(z+h/2)\}]\partial/\partial z\right)G_0(z,z') = -\delta(z,z')\,.$$

The coupling of the guided modes with this radiative mode is then calculated by overlap integrals

$$\hat{C}_{\text{rad}} = I_0 \begin{pmatrix} 1 & 1/2 & -1/2 & -1 & -1/2 & 1/2 \\ 1/2 & 1 & 1/2 & -1/2 & -1 & -1/2 \\ -1/2 & 1/2 & 1 & 1/2 & -1/2 & -1 \\ -1 & -1/2 & 1/2 & 1 & 1/2 & -1/2 \\ -1/2 & -1 & -1/2 & 1/2 & 1 & 1/2 \\ 1/2 & -1/2 & -1 & -1/2 & 1/2 & 1 \end{pmatrix},$$

where

$$I_0 = \kappa_1^2 \iint_{PC} \{[-\partial^2/\partial z^2 + [\delta(z-h/2) - \delta(z+h/2)]\partial/\partial z] \cdot G_0(z,z') \\ [(\partial^2/\partial z'^2 - [\delta(z'-h/2) - \delta(z'+h/2)]\partial/\partial z')\Theta_0(z')]\}\Theta_0^*(z)dz'dz \quad (57)$$

is a purely imaginary quantity.

To block-diagonalize $\hat{C}$, we next perform the unitary transformation $\hat{C}_{\text{cp}} = U\hat{C}U^{-1}$ with the matrix

$$U = \frac{1}{\sqrt{6}} \begin{pmatrix} 1 & 1 & 1 & 1 & 1 & 1 \\ 1 & e^{i\pi/3} & e^{2i\pi/3} & -1 & e^{-2i\pi/3} & e^{-i\pi/3} \\ 1 & e^{2i\pi/3} & e^{-2i\pi/3} & 1 & e^{2i\pi/3} & e^{-2i\pi/3} \\ 1 & e^{-i\pi/3} & e^{-2i\pi/3} & -1 & e^{2i\pi/3} & e^{i\pi/3} \\ 1 & e^{-2i\pi/3} & e^{2i\pi/3} & 1 & e^{-2i\pi/3} & e^{2i\pi/3} \\ 1 & -1 & 1 & -1 & 1 & -1 \end{pmatrix}.$$

After excluding the first and the sixth rows describing singlet states, we get the matrix in the subspace of the circular-polarized states.

It can be proven using Sup. Eq. (53) that in the vicinity of the Γ point, the propagation

constants of the modes are linearly dependent on small deviations (k_x, k_y) [11]

$$\beta_j = \beta_0 + \tilde{\beta}\left(\frac{G_{jx}}{G}k_x + \frac{G_{jy}}{G}k_y\right), \tag{58}$$

that is the right-hand side of Sup. Eq. (54) is additionally corrected with the diagonal matrix $\Delta\hat{K}$

$$\Delta\hat{K} = 2\beta_0\tilde{\beta}\begin{pmatrix} k_y & 0 & 0 & 0 & 0 & 0 \\ 0 & \sqrt{3}k_x/2 + k_y/2 & 0 & 0 & 0 & 0 \\ 0 & 0 & \sqrt{3}k_x/2 - k_y/2 & 0 & 0 & 0 \\ 0 & 0 & 0 & -k_y & 0 & 0 \\ 0 & 0 & 0 & 0 & -\sqrt{3}k_x/2 - k_y/2 & 0 \\ 0 & 0 & 0 & 0 & 0 & \sqrt{3}k_x/2 + k_y/2 \end{pmatrix}.$$

As a result, we finally obtain the right-hand side of the eigenvalue problem Sup. Eq. (54) describing a photonic bandstructure of the PhC slab in the proximity of Γ point as follows

$$\hat{C}_{\text{cp}} + \Delta\hat{K}_{\text{cp}} = \begin{pmatrix} (\kappa_1 - \kappa_2 - \kappa_3)G^2 & -\beta_0\tilde{\beta}(ik_x - k_y) & 0 & 0 \\ \beta_0\tilde{\beta}(ik_x + k_y) & (\kappa_3 - \kappa_2 - \kappa_1)G^2 & 0 & 0 \\ 0 & 0 & (\kappa_1 - \kappa_2 - \kappa_3)G^2 & \beta_0\tilde{\beta}(ik_x + k_y) \\ 0 & 0 & -\beta_0\tilde{\beta}(ik_x - k_y) & (\kappa_3 - \kappa_2 - \kappa_1)G^2 \end{pmatrix}$$
$$+ I\begin{pmatrix} \frac{\kappa_1 + \kappa_2}{2} + \kappa_3 & 0 & 0 & 0 \\ 0 & \frac{\kappa_2 - \kappa_1}{2} - \kappa_3 & 0 & 0 \\ 0 & 0 & \frac{\kappa_1 + \kappa_2}{2} + \kappa_3 & 0 \\ 0 & 0 & 0 & \frac{\kappa_2 - \kappa_1}{2} - \kappa_3 \end{pmatrix} + I_0\begin{pmatrix} 3 & 0 & 0 & 0 \\ 0 & 0 & 0 & 0 \\ 0 & 0 & 3 & 0 \\ 0 & 0 & 0 & 0 \end{pmatrix}, \tag{59}$$

where similarly to Sections I and II we redefine $k_x \to k_y$, $k_y \to k_x$. In the absence of dissipation, the first and second matrices in Sup. Eq. (59) compose the Hermitian Hamiltonian which contains the Dirac part and real mass-terms. The imaginary part of p (dipolar) modes eigenfrequencies is

determined by I_0 which is directly related to the magnitude of radiative losses γ_r. Thus, the GME method confirms applicability of our phenomenological model.

Supplementary Note 5. SOS (Silicon on Sapphire) implementation of the metasurface. Fabrication Recipe

Supplementary Figure 6 illustrates the main stages of the technique used for the metasurface fabrication.

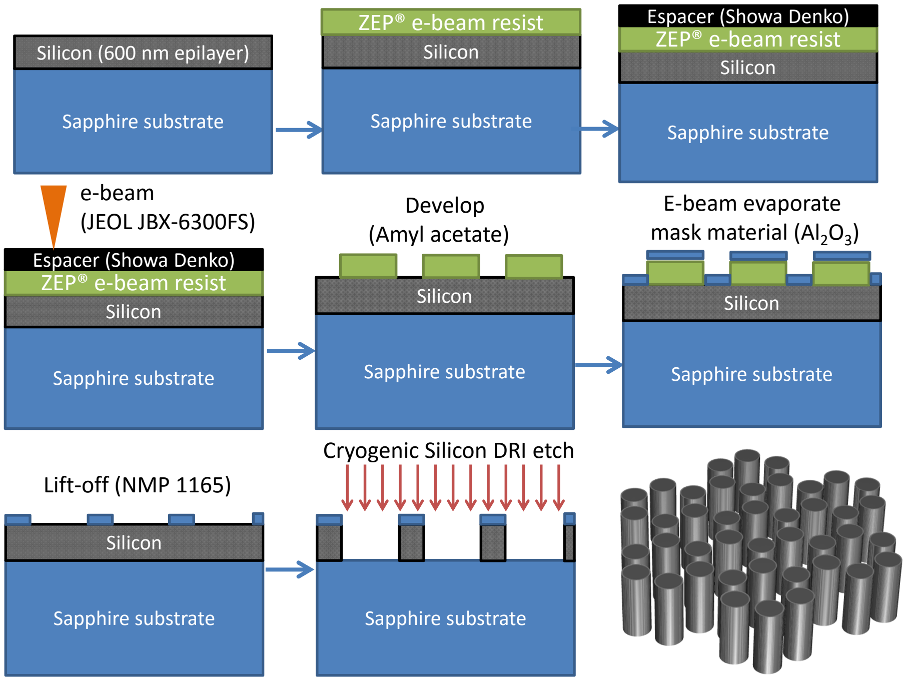

Supplementary Figure 6. Schematic of the metasurface fabrication procedure.

Supplementary Note 6. Coupled mode theory for the metagrating based on tight binding approach

To describe the excitation of the edge states on a sample consisting of an array of domain walls formed by repetitive stitching of N unit cells of shrunken and expanded domains, we use coupled mode theory (CMT) described in Supplementary Note 1 with the Hamiltonian derived from the tight-binding method. The intercell and intracell tunneling amplitudes are denoted as J and K, respectively. The translation vectors of the lattice are equal to $\mathbf{a}_1 = a\ (1, 0)$ and $\mathbf{a}_2 = a\ \left(1/2, \sqrt{3}/2\right)$, where a is the lattice period. Bloch vectors are defined as $k_p = 2\pi/\lambda \sin(\theta)$, where λ is the wavelength of the incident plane wave, and θ is the angle between the propagation direction and normal to the sample. The propagation plane is chosen to be x-z, and the electric field is polarized along y direction. Due to symmetry matching, only dipolar modes are directly excited by the plane wave source. Two types of dipolar modes can be excited at small incidence angles $\theta \approx 0$, namely $|U_1\rangle = (1, 1, 0, -1, -1, 0)^T$ and $|U_2\rangle = (1, 0, -1, -1, 0, 1)^T$, which are eigenmodes of tight-binding unit cell Hamiltonian near normal incidence angle. Since the polarization of the impinging wave is fixed, we consider only one of the dipolar modes, specifically, $|U_1\rangle$ here; excitation of the mode $|U_2\rangle$ can be described by the same method. Domain walls with zigzag cuts are aligned along the x direction. Thus, the system is described by the following equations:

$$-\,i\,\varepsilon\ |\psi\rangle = -i\,\hat{H}\ |\psi\rangle + \varkappa\,\hat{S}_1 - \hat{L}\ |\psi\rangle\ , \qquad (60)$$

where Hamiltonian of the meta-grating structure $\hat{H}$ is represented as a direct sum of the Hamiltonians corresponding to the strips of shrunken or expanded structures, and it is derived from tight

binding model which reads

$$\begin{aligned}\hat{H} &= \mathrm{Diag}\,(N,0)\otimes\hat{H}_I\oplus\mathrm{Diag}\,(N,0)\otimes\hat{H}_{II}\\ &+\mathrm{Diag}\,(N,-1/2)\otimes(\hat{H}_{\mathrm{m1}}\oplus\hat{H}_{\mathrm{m2}})+\mathrm{Diag}\,(N,1/2)\otimes(\hat{H}_{\mathrm{p1}}\oplus\hat{H}_{\mathrm{p2}})\\ &+\mathrm{Diag}\,(2N,-2)\otimes\hat{H}_{\mathrm{mm}}+\mathrm{Diag}\,(2N,2)\otimes\hat{H}_{\mathrm{pp}}.\end{aligned}\tag{61}$$

Here $\mathrm{Diag}\,(N,n)$ means $N\times N$ n^{th} diagonalized matrix and with unity elements 1, and $\hat{H}_{I/II}$, $\hat{H}_{\mathrm{p}1,2/\mathrm{m}1,2}$ and $\hat{H}_{\mathrm{pp/mm}}$ are defined as

$$\hat{H}_I = \begin{pmatrix} 0 & -K & 0 & 0 & 0 & -K \\ -K & 0 & -K & 0 & 0 & 0 \\ 0 & -K & 0 & -K & 0 & ,0 \\ 0 & 0 & -K & 0 & -K & 0 \\ 0 & 0 & 0 & -K & 0 & -K \\ -K & 0 & 0 & 0 & -K & 0 \end{pmatrix}, \hat{H}_{II} = \hat{H}_I\,(K \leftrightarrow J)\,, \tag{62}$$

$$\hat{H}_{\mathrm{m}1} = \begin{pmatrix} 0 & 0 & 0 & 0 & 0 & 0 \\ 0 & 0 & 0 & 0 & 0 & 0 \\ 0 & 0 & 0 & 0 & 0 & -J \\ 0 & 0 & 0 & 0 & 0 & 0 \\ 0 & e^{-i\sqrt{3}k_x a} & 0 & 0 & 0 & 0 \\ 0 & 0 & 0 & 0 & 0 & 0 \end{pmatrix}, \hat{H}_{\mathrm{p}1} = \begin{pmatrix} 0 & 0 & 0 & 0 & 0 & 0 \\ 0 & 0 & 0 & 0 & -J & 0 \\ 0 & 0 & 0 & 0 & 0 & 0 \\ 0 & 0 & 0 & 0 & 0 & 0 \\ 0 & 0 & 0 & 0 & 0 & 0 \\ 0 & 0 & e^{-i\sqrt{3}k_x a} & 0 & 0 & 0 \end{pmatrix}, \tag{63}$$

$$\hat{H}_{\mathrm{m}2} = \bar{\hat{H}}_{\mathrm{p}1}, \hat{H}_{\mathrm{p}2} = \bar{\hat{H}}_{\mathrm{m}1}, \tag{64}$$

$$\hat{H}_{\mathrm{mm}} = \begin{pmatrix} 0 & 0 & 0 & 0 & 0 & 0 \\ 0 & 0 & 0 & 0 & -J & 0 \\ 0 & 0 & 0 & 0 & 0 & 0 \\ 0 & 0 & 0 & 0 & 0 & 0 \\ 0 & 0 & 0 & 0 & 0 & 0 \\ 0 & 0 & e^{-i\sqrt{3}k_x a} & 0 & 0 & 0 \end{pmatrix}, \hat{H}_{\mathrm{pp}} = \bar{\hat{H}}_{\mathrm{mm}}. \tag{65}$$

The source matrix is $\hat{S}_1 = E_{\text{in}}\,|2N\rangle \otimes |U_1\rangle$, where $|2N\rangle = (1, 1, ..., 1)^T$, which has $2N$ sites, and the loss matrix L containing both internal loss γ_0 and radiative loss $\varkappa^2/2$ has the form

$$\hat{L} = \text{Diag}\,(2N, 2N) \otimes \begin{pmatrix} \gamma_0 + \varkappa^2/2 & 0 & 0 & 0 & 0 & 0 \\ 0 & \gamma_0 + \varkappa^2/2 & 0 & 0 & 0 & 0 \\ 0 & 0 & \gamma_0 & 0 & 0 & 0 \\ 0 & 0 & 0 & \gamma_0 + \varkappa^2/2 & 0 & 0 \\ 0 & 0 & 0 & 0 & \gamma_0 + \varkappa^2/2 & 0 \\ 0 & 0 & 0 & 0 & 0 & \gamma_0 \end{pmatrix} \tag{66}$$

Periodic boundary conditions are applied at the outer domain walls in the y direction. Solving Sup. Eq. (60), transmittance and extinction can be calculated similar to Sup. Eq. (47).

Compared to the bulk structures without domain walls, the extra periodicity of domain walls along y direction with the lattice constant $a_y = \sqrt{3}Na_0$ supplies extra diffraction channels for the scattered fields in the wavelength range under study if the incidence angle is swept along y direction. Due to the continuity of E_y at boundaries $y = na_y$, the transmitted fields can be written as [12]

$$E_y^t(\mathbf{r}) = \sum_{m=-\infty}^{\infty} \sum_{n=1}^{\infty} a_{n,m} e^{i(k_x^m x + k_z z)} \sin k_y^n y \tag{67}$$

where $k_x^m = 2\pi\, m/a$, $k_y^n = 2\pi/\lambda \sin(\theta) + n\pi/a_y$, and $k_z = \sqrt{(2\pi/\lambda\, n_{\text{eff}})^2 - [k_x^m]^2 - [k_y^m]^2}$, n_{eff} is the effective index of the substrate. We notice these diffraction modes become leaky for $k_z > 0$, thus both reflectivity and transmittance drop due to coupling of the leaky modes to the internal modes of the structure.

Supplementary Note 7. The role of non-Hermiticity

Radiative and non-radiative losses can be included explicitly into the system Hamiltonian. As a result, the obtained effective Hamiltonian will be non-Hermitian. For instance, the expression for the block $\hat{H}_-$ describing one of the pseudospins reads:

$$\hat{H}_-^{\mathrm{eff}}(\mathbf{k}) = v\,[\sigma_x\,k_x + \sigma_y\,k_y] + [\mu(\mathbf{k}) - i\,\gamma_{\mathrm{r}}/2]\;\sigma_z - i\,(\gamma_0 + \gamma_{\mathrm{r}}/2)\,\hat{I}\;. \tag{68}$$

In analogy with the standard derivation [4] it can be verified that despite the losses the boundary of the two materials with the opposite sign of effective mass μ still supports the edge mode. The energy of this mode becomes complex due to the losses:

$$\varepsilon = v\,k_y - i\,(\gamma_0 + \gamma_{\mathrm{r}}/2)\;, \tag{69}$$

where we assume that the boundary of the two materials is along the y-axis. Equation (69) means that the edge mode has a finite lifetime $\tau = (\gamma_0 + \gamma_{\mathrm{r}}/2)^{-1}$. However, if the system is pumped externally, the edge mode still has the impact on the metasurface scattering characteristics. The two components of the Dirac spinor $|\psi\rangle$ are described by $\psi_{1,2}(x) = A_{1,2}\,e^{\lambda\,x}$ for $x < 0$ and $\psi_{1,2}(x) = A_{1,2}\,e^{-\lambda\,x}$ for $x > 0$. Parameter λ acquires a non-zero imaginary part:

$$\lambda = \frac{\mu(\mathbf{k}) - i\,\gamma_{\mathrm{r}}/2}{v}\;, \tag{70}$$

i.e. losses introduce an oscillatory behavior of the eigenfunctions.

In the non-Hermitian case left $\left|\psi^L\right\rangle$ and right $\left|\psi^R\right\rangle$ eigenvectors of the Hamiltonian do not coincide which leads to the four possibilities of the Berry connection definition: $A_m^{LL}(\mathbf{k}) = i\,\left\langle\psi^L\right|\partial_m\psi^L\rangle$, $A_m^{LR}(\mathbf{k}) = i\,\left\langle\psi^L\right|\partial_m\psi^R\rangle$, $A_m^{RL}(\mathbf{k}) = i\,\left\langle\psi^R\right|\partial_m\psi^L\rangle$, and $A_m^{RR}(\mathbf{k}) = i\,\left\langle\psi^R\right|\partial_m\psi^R\rangle$, where ∂_m means here $\partial/\partial\,k_m$. Though these four definitions have a different form, we confirmed

that for the parameters corresponding to the metasurface studied here, they yield the same result for the spin-Chern number [13]. Thus, as evidenced by both theory and experiment, the edge states still persist in the presence of losses, and in our case they can be conceived from the spin Chern number of bulk states.

References

1. Wu, L.-H. & Hu, X. Scheme for Achieving a Topological Photonic Crystal by Using Dielectric Material. *Physical Review Letters* **114**, 23901 (2015).

2. Barik, S., Miyake, H., DeGottardi, W., Waks, E. & Hafezi, M. Two-dimensionally confined topological edge states in photonic crystals. *New Journal of Physics* **18**, 113013 (2016).

3. Bir, G. L. & Pikus, G. E. *Symmetry and strain-induced effects in semiconductors* (Keter Publishing Hourse, Jerusalem, 1974).

4. Shen, S.-Q. *Topological Insulators. Dirac Equation in Condensed Matters* (Springer, Heidelberg, 2012).

5. Raghu, S. & Haldane, F. D. M. Analogs of quantum-Hall-effect edge states in photonic crystals. *Physical Review A* **78**, 033834 (2008).

6. Khanikaev, A. B. *et al.* Photonic topological insulators. *Nature Materials* **12**, 233-239 (2013).

7. Haus, H. A. *Waves and Fields in Optoelectronics* (Prentice-Hall, Englewood Cliffs, New Jersey, 1984).

8. Suh, W., Wang, Z. & Fan, S. Temporal Coupled-Mode Theory and the Presence of Non-Orthogonal Modes in Lossless Multimode Cavities. *IEEE Journal of Quantum Electronics* **40**, 1511–1518 (2004).

9. Andreani, L. C. & Gerace, D. Photonic-crystal slabs with a triangular lattice of triangular holes investigated using a guided-mode expansion method. *Physical Review B* **73**, 235114 (2006).

10. Yang, Y., Peng, C., Liang, Y., Li, Z. & Noda, S. Three-dimensional coupled-wave theory for the guided mode resonance in photonic crystal slabs: TM-like polarization. *Optics Letters* **39**, 4498–4501 (2014).

11. Yin, X. *et al.* Analytical study of mode degeneracy in non-Hermitian photonic crystals with TM-like polarization. *Physical Review B* **96**, 075111 (2017).

12. Sakoda, K. *Optical Properties of Photonic Crystals* (Springer, Berlin, 2005).

13. Shen, H., Zhen, B., & Fu, L. Topological Band Theory for Non-Hermitian Hamiltonians. *Preprint at http://arxiv.org/abs/1706.07435* (2017).
